# It Takes Two to Make a Thing Go Right: Epistasis, Two-Component Response Systems, and Bacterial Adaptation

**DOI:** 10.3390/microorganisms12102000

**Published:** 2024-09-30

**Authors:** Brittany R. Sanders, Lauren S. Thomas, Naya M. Lewis, Zaria A. Ferguson, Joseph L. Graves, Misty D. Thomas

**Affiliations:** Department of Biology, North Carolina Agricultural and Technical State University, Greensboro, NC 27411, USA; brsander@ncat.edu (B.R.S.); lauren_thomas@brown.edu (L.S.T.); naya.m.lewis@vanderbilt.edu (N.M.L.); zferguson5@student.gsu.edu (Z.A.F.);

**Keywords:** epistasis, bacterial adaptation, two-component response systems, silver resistance, gene-by-environment interactions

## Abstract

Understanding the interplay between genotype and fitness is a core question in evolutionary biology. Here, we address this challenge in the context of microbial adaptation to environmental stressors. This study explores the role of epistasis in bacterial adaptation by examining genetic and phenotypic changes in silver-adapted *Escherichia coli* populations, focusing on the role of beneficial mutations in two-component response systems (TCRS). To do this, we measured 24-hour growth assays and conducted whole-genome DNA and RNA sequencing on *E. coli* mutants that confer resistance to ionic silver. We showed recently that the R15L *cusS* mutation is central to silver resistance, primarily through upregulation of the *cus* efflux system. However, here we show that this mutation’s effectiveness is significantly enhanced by epistatic interactions with additional mutations in regulatory genes such as *ompR*, *rho*, and *fur*. These interactions reconfigure global stress response networks, resulting in robust and varied resistance strategies across different populations. This study underscores the critical role of epistasis in bacterial adaptation, illustrating how interactions between multiple mutations and how genetic backgrounds shape the resistance phenotypes of *E. coli* populations. This work also allowed for refinement of our model describing the role TCRS genes play in bacterial adaptation by now emphasizing that adaptation to environmental stressors is a complex, context-dependent process, driven by the dynamic interplay between genetic and environmental factors. These findings have broader implications for understanding microbial evolution and developing strategies to combat antimicrobial resistance.

## 1. Introduction

Understanding the relationships between genotype and fitness is a fundamental question in evolutionary biology, especially in the context of microbial adaptation to environmental stressors [1,2,3]. A key aspect of this understanding is the study of epistasis, which refers to interactions between different genetic loci and their combined effect on an organism’s fitness. Epistatic interactions are crucial for revealing the complexity of adaptive evolution, as they can significantly influence the trajectory of evolutionary changes in microbial populations [4,5]. Fitness epistasis highlights different types of genetic interactions—whether positive, negative, or sign epistasis—can alter the expected outcomes of adaptive mutations, making it a vital consideration in the study of complex traits [4,6,7,8]. 

Historically, silver has been used for centuries in antimicrobial applications due to its broad-spectrum antibacterial activity. Silver has been shown to impact disruption of the cell wall and membrane; interact with the thiol groups of respiratory enzymes; disrupt respiration due to binding to the cell membrane; disrupt the uptake of phosphorus and cause release of phosphates, mannitol, succinate, proline, and glutamine; disrupt metabolism, cell signaling, DNA replication, transcription, translation, and cell division, either directly or through the generation of reactive oxygen species (ROS). This makes it a valuable tool in preventing infections and preserving materials [9,10,11]. However, the emergence of silver-resistant bacteria poses a significant challenge to its effectiveness. The rising prevalence of silver resistance is not only a concern in clinical settings but also has broader implications for environmental health and the efficacy of silver-based antimicrobials [12,13,14,15].

The adaptive response of bacteria to such environmental stressors is often mediated by sophisticated regulatory networks, including two-component response systems (TCRS) [16,17,18]. TCRS are widespread in bacteria and play a pivotal role in sensing and responding to environmental changes. These systems typically consist of a sensor kinase and a response regulator that work together to modulate gene expression in response to specific stimuli [19,20,21]. In particular, TCRS like the CusS/CusR system are essential for metal ion homeostasis, regulating the efflux of toxic ions such as copper and silver, and thus play a central role in bacterial survival under metal stress [22,23,24].

The interplay between two-component response systems (TCRS) and epistasis is particularly significant in the context of bacterial resistance to antimicrobial agents [25,26,27]. When adaptive mutations occur within TCRS genes, these mutations do not act in isolation; rather, their phenotypic effects are often modulated by the genetic background of the organism and the specific environmental conditions in which the bacteria find themselves. This interaction can lead to complex and sometimes unpredictable adaptive responses [25]. Furthermore, epistasis can further complicate the outcome of adaptive mutations within TCRS. A mutation that increases resistance in one genetic background may have a different effect, or even an opposite effect, in another, depending on the presence of other interacting mutations [4,8,28,29]. Moreover, epistasis can also influence protein–protein interactions within TCRS and can result in varying degrees of resistance, depending on the combination of mutations and environmental pressures [27]. This suggests that the adaptive landscape of TCRS is highly contingent on both the internal genetic network and external environmental factors, making the evolution of resistance a dynamic process, and this complexity underscores the challenges in predicting bacterial resistance [30,31,32].

Experimental evolution has become a powerful approach to studying these processes, allowing researchers to observe evolutionary changes in real-time and identify adaptive mutations through techniques such as whole-genome resequencing [2,33,34]. Several studies have employed these methods to evaluate the potential for silver resistance in *Escherichia coli* and found that several mutations, namely, *cusS, ompR, rpoB,* and *purL,* were associated with the adaptive response [35,36,37]. We then recently demonstrated that specific mutations in the *cusS* gene could drive significant changes in gene expression and bacterial fitness, particularly in response to silver stress [38]. However, these mutations alone were insufficient to fully explain the observed resistance in the past studies, suggesting that additional genetic interactions were at play. We therefore hypothesized that these *cusS* mutations interact with other genetic changes via positive epistasis to enhance the overall resistance phenotype. 

Given this context, our current study aims to further explore the role of epistasis in bacterial adaptation by examining the interplay between *cusS* mutations and other genetic changes within silver-adapted *Escherichia coli* populations. Specifically, we seek to answer the following key questions:

1. How do *cusS* mutations, in combination with other genetic changes, influence the overall fitness and resistance of *E. coli* to silver?
2. What are the specific gene expression patterns associated with these mutations, and how do they contribute to the observed phenotypes?
3. How do epistatic interactions among these mutations shape the evolutionary trajectory of the bacteria in response to silver stress?
4. How does our model of TCRS-driven bacterial adaptation need to be refined in light of these findings?

To address these questions, we employed a combination of 24-hour growth assays along with whole-genome DNA and RNA sequencing to systematically analyze the phenotypic and genetic characteristics of the silver-adapted mutants. This approach allowed us to dissect the complex genetic interactions that underpin silver resistance in these bacterial populations and to better describe the epistatic interactions that complement adaptive mutations in TCRS genes.

## 2. Materials and Methods

### 2.1. Bacterial Strains and Culturing

*Escherichia coli* K-12 MG1655 (ATCC #10798D-5, Manassas, VA, USA) was used as our wild-type (WT) strain, as it served as the ancestral strain in the experimental evolution (EE) studies where our mutants were initially identified [36,37]. The archived stocks of the silver-adapted mutants (SAMs) were revived from storage at −80 °C. Of the original 16 strains, only 7 were successfully recovered during the revival process, and these populations have been designated as SAM1-7. We previously published the design, construction, and whole genome sequencing of our single *cusS* variants (R15L, T14P, T17P, N279H) [39], which were utilized again in this study. All growth experiments were conducted in Davis Minimal Broth (DMB—7 g/L dipotassium phosphate, 2 g/L monopotassium phosphate, 0.5 g/L sodium citrate, 1 g/L ammonium sulfate, and 0.1 g/L magnesium sulfate, ThermoFisher, Waltham, MA, USA) supplemented with 10% dextrose as a carbon source, incubated overnight at 37 °C with continuous shaking at 200 rpm, unless otherwise noted. DMB was selected as it was the original medium used during the silver adaptation study, where these mutants had initially evolved.

### 2.2. Whole Genome Illumina Sequencing of the SAM Populations 

Bacterial stocks from the silver-adapted mutant (SAM) populations in the Tajkarimi et al., 2017 [37] EE study were grown overnight in DMB, pelleted, and sent for whole genome Illumina sequencing at the Sequencing Center (SeqCenter.com) in Pittsburgh, PA on an Illumina NovaSeq X Plus (San Diego, CA, USA). Sequence alignment and variant calling were performed using the breseq 0.30.0 pipeline [40], with alignment to the *E. coli* K-12 MG1655 reference sequence (NC_000913). The original WT strain was sequenced in one of our previous studies [36]. It is important to note that after DNA sequencing, the only *cusS* mutation detected in the SAM populations was R15L. As a result, this was the only single adaptive mutant used for the remainder of the study and will be referred to simply as R15L from this point forward. Whole genome sequencing fastq files are available in the NCBI SRA database using the BioProject ID: PRJNA1160277 and BioSamples: SAMN43760522-SAMN43760542.

### 2.3. RNAseq and Differential Gene Expression 

Overnight cultures of the WT and each mutant population (R15L and SAM1-7) were grown and then subcultured at a 1:100 ratio in DMB (6 replicates). These cultures were grown to an OD_600_ of 0.2, as this was where our populations reach mid-log phase when grown in DMB. Once the desired optical density was reached, three replicates of each culture were incubated for 1 h at 37 °C, then pooled, pelleted, and stored at −80 °C. The remaining three replicates were exposed to 50 ng/mL silver nitrate (the original selection concentration) for 1 h at 37 °C, then pooled, pelleted, and stored at −80 °C. The frozen bacterial pellets were shipped to SeqCenter (seqcenter.com). At SeqCenter, samples were treated with Invitrogen DNase (RNase-free) (Waltham, MA, USA) and RNA extractions were performed. Library preparation was conducted using Illumina’s Stranded Total RNA Prep Ligation with Ribo-Zero Plus kit and 10 bp IDT for Illumina indices. Sequencing was performed on a NextSeq 2000, generating 2 × 51 bp reads. Demultiplexing, quality control, and adapter trimming were performed using bcl-convert (v3.9.3) [41]. The average reads per sample exceeded 20 million, with over 94% of bases having a quality score greater than Q30. Read mapping was carried out with HISAT2 (v2.2.0) [42], and read quantification was performed using Subread’s featureCounts (v2.0.1) functionality. The read counts were loaded into R (4.0.2) and normalized using edgeR’s (v1.14.5) Trimmed Mean of M values (TMM) algorithm [43]. Subsequent values were converted to counts per million (CPM). Differential expression analysis was performed using edgeR’s exact test to compare differences between two groups of negative-binomial counts, with an estimated dispersion value of 0.1. All populations were compared to the WT in the absence of silver nitrate for this analysis. Individual genes were then grouped into categories based on their biological function using a combination of literature searchers and databases such as UniProt, KEGG, and Gene Ontology. RNAseq fastq files are available in the NCBI SRA database using the BioProject ID: PRJNA1160277 and BioSamples: SAMN43760522-SAMN43760542.

### 2.4. Twenty-Four-Hour Growth Assays 

The 24-hour growth assays were conducted using the WT, R15L, and SAM1-6 (SAM7 was lost after sequencing and could not be included) to evaluate changes in bacterial growth and determine minimum inhibitory concentrations (MICs) in the presence of increasing concentrations of silver nitrate (0–750 ng/mL). To begin, archived glycerol stocks of each population were inoculated in DMB and incubated overnight at 37 °C with continuous shaking at 200 rpm. The following day, overnight cultures were diluted to an OD_600_ of 0.05 for normalization and added (in triplicate) to a 96-well plate containing a gradient of silver nitrate concentrations. The 96-well plates were then covered with optically clear MicroAmp Adhesive film (ThermoFisher, Waltham, MA, USA) and absorbance readings were taken every hour from 0 to 24 h using a pre-programmed protocol on the Varioskan Lux 96-well plate reader (ThermoFisher, Waltham, MA, USA) at 37 °C with shaking. To normalize the data, blank readings (in triplicate) were averaged, and the mean values were subtracted from each replicate population at each concentration. The resulting data were plotted using GraphPad Prism version 8.0.0 for Mac OS X. We then used the Growthcurver package (v0.3.1) in R to fit our growth curve data for each population at each concentration of silver nitrate to a standard form of the logistic equation [44]. This enabled us to calculate the initial population size (n0), the carrying capacity (k), the growth rate (r), the time to midpoint (t_mid), the generation time (t_gen), the area under the curve—log phase (auc_l) and the goodness-of-fit (sigma). The (k), (r), (t_mid) and (t-gen) were plotted in GraphPad Prism. MICs were determined by identifying the lowest concentration at which no growth was observed for each population and are reported on their corresponding curve. Growth curves were also used as the basis for calculating the relative fitness. The calculation for relative fitness (ω) was performed by taking the OD value for each population at a specific time point and dividing it by the OD value of the fittest population at that same time point. These data were then plotted in Prism, and one-way ANOVAs with multiple pairwise comparisons were used to determine statistical differences.

## 3. Results

### 3.1. DNA Sequencing and Mutation Analysis 

Populations carried variation in the number of total mutations, but most notably the R15L *cusS* mutation was found in SAM2, SAM3, SAM6, and SAM7, all to fixation (*f* = 1.00). All moderate and high frequency mutations are given in Table 1 and a complete list of all mutations can be found in Table S1.This mutation was the most common in the original evolution study and the only one revivable from storage [37]. Notably, the R15L mutation was consistently associated with additional mutations in other key regulatory genes, such as *rho* and *ompR* in all four of these populations. SAM2 and SAM3 fixed an R66C mutation in *rho* and a 1 bp deletion in *ompR*, while SAM6 and SAM7 fixed G63V in *rho* and R182C in *ompR*. These R15L populations then diverged, with SAM2 and SAM3 sharing additional mutations in *dnaK*, *yggN*, *yghS*, and *yicO*, in addition to a 103 bp deletion in *yicC*, and SAM6 and SAM7 both carrying K9N in *fur* and V282L in *rpo*. SAM7 also uniquely fixed three intergenic mutations. Overall, SAM2 had 39 mutations, SAM3 had 24, SAM6 had 35, and SAM7 had 17. SAM1, SAM4, and SAM5 lacked *cusS* mutations or did not reach fixation (SAM4 had a *cusS* mutation with an *f* = 0.055). SAM1 fixed A17V in *glnH*, while SAM4 and SAM5 had no fixed mutations. SAM4’s highest was an 82 bp deletion in *rph* (*f* = 0.533), and SAM5’s was E783V in *glnE* (*f* = 0.408). In total, SAM1 carried 17 mutations, SAM4 had 12, and SAM5 had 17—generally fewer overall than the R15L populations. This suggests that the *cusS* mutation may be crucial for driving the selection and fixation of other genes in response to silver nitrate, though *cusS* alone is not enough for full resistance [38].

### 3.2. Gene Expression and Functional Implications 

**Figure 1:**
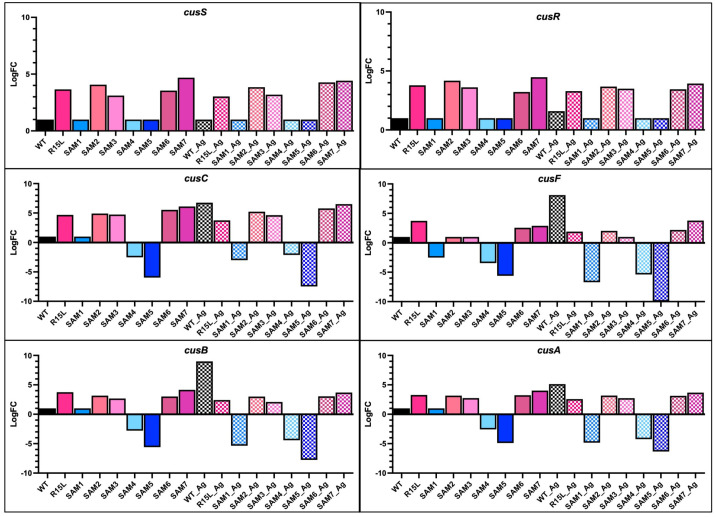
Differential expression of *cus* genes across silver-adapted *E. coli* populations: This figure illustrates the expression levels of the *cusS/R* two-component response system (TCRS) genes and the *cusCFBA* efflux genes, which are essential for silver and copper ion efflux, across various silver-adapted *E. coli* populations in both the presence (checkered bars) and absence of silver nitrate (solid bars). All expression levels are normalized to the wild-type (WT) in the absence of silver nitrate, where WT is assigned a log fold change (logFC) of 1. LogFC values were plotted using GraphPad Prism. The upregulation observed in populations with the R15L mutation in *cusS* highlights its role in enhancing the efficiency of the efflux system, thereby increasing silver resistance. This figure also demonstrates the variation in gene expression across different populations, reflecting their differing capacities to manage metal ion toxicity. Notably, SAM populations that do not carry the R15L mutation exhibit no expression in the *cusS/R* genes and downregulate the efflux pump genes, indicating a distinct response mechanism.

We previously associated the R15L mutation in *cusS* with significant upregulation and constitutive expression of the *cusCFBA* operon, which is central to the bacterial response to silver stress [38]. CusC is a component of the outer membrane porin channel, CusB serves as a membrane fusion protein, CusA is the inner membrane component that actively exports silver and copper ions, and CusF is a periplasmic metallochaperone [45,46]. The increased expression enhances the efflux system’s efficiency, contributing to a robust silver resistance phenotype. RNAseq analysis here revealed distinct gene expression profiles in these efflux genes across the SAM mutants compared to the WT strain and single R15L mutant (Figure 1). 

**Figure 2:**
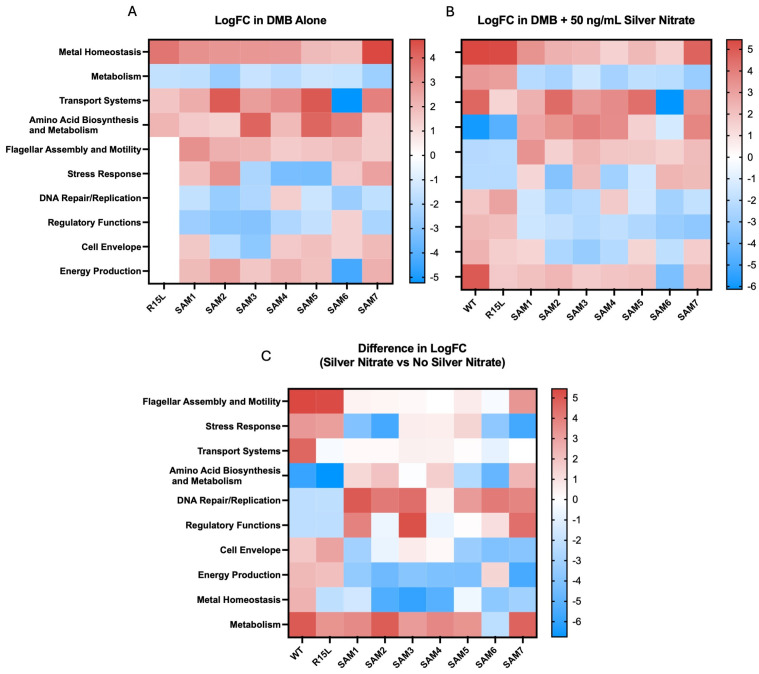
Comparative gene expression across 10 biological categories in silver-adapted *E. coli* populations: Heatmaps illustrate differential expression of genes across 10 key biological categories in various silver-adapted *E. coli* populations, normalized to the wild-type (WT) in the absence of silver nitrate. Differentially expressed genes were categorized based on their biological functions, and the heatmaps display the averaged differential expression for all genes within each category. Warmer colors indicate higher expression levels, while cooler colors indicate lower expression levels. The heatmaps were generated using GraphPad Prism. Subfigure (**A**) shows gene expression in the absence of silver nitrate, serving as a baseline to display natural variations in gene regulation and adaptation strategies across different populations. Subfigure (**B**) highlights gene expression in the presence of silver nitrate. Subfigure (**C**) is a difference map illustrating the changes in gene expression between conditions with and without silver nitrate, providing a direct comparison of how silver exposure affects gene expression across different biological categories. These heatmaps collectively emphasize how specific genetic backgrounds modulate these adaptive responses to silver exposure.

The non-R15L-carrying mutants like SAM1 showed only moderate upregulation in metal homeostasis genes (Figure 2) such as *zntA* and *cueO* and all down regulated expression of all of the *cus* efflux pump genes in both the presence and absence of silver nitrate (Figure 1). While all SAM mutants carrying the R15L mutation demonstrated upregulation in metal homeostasis genes, the extent of this response varied (Figure 2). Specifically, they all exhibited constitutive upregulation in both *cusS/R* and *cusCFBA* (Figure 1). SAM7 exhibited the highest overall upregulation in the *cusCFBA* efflux genes, suggesting that additional mutations in its genome, particularly those in *rho* and *ompR*, might enhance its ability to manage metal ion stress. Clearly, the R15L mutation in *cusS* does not act in isolation, as the single mutant (R15L) displays lower expression levels than the SAMs. This supports the idea that expression of the *cusCFBA* efflux pump genes is enhanced by the other mutations observed in the genetic background of the SAM populations. Providing evidence of positive epistasis suggests that the regulatory disruptions caused by the additional mutations may facilitate a more robust and sustained activation of the *cus* operon, leading to heightened silver resistance. 

Beyond the *cus* operon, differential gene expression analysis revealed several other biological pathways significantly altered in response to silver adaptation across the SAM mutants (Figure 2):

1. Oxidative Stress Response Genes: Many SAM mutants, particularly those with *cusS* mutations, showed upregulation in genes associated with the oxidative stress response. For instance, *msrP* (methionine sulfoxide reductase) and *huiH* (hypothetical protein involved in stress response) were consistently upregulated. Also, *msrP* is known for its role in reducing methionine sulfoxide residues in proteins, which helps protect cells from oxidative damage caused by reactive oxygen species (ROS) [47]. The upregulation of these genes suggests that the mutants have developed a preemptive defense mechanism to mitigate oxidative damage associated with silver ion exposure, contributing to their enhanced survival under stress.
2. Transport Systems Genes: The expression of various transport-related genes, beyond the *cus* operon, was significantly altered. SAM2 and SAM5, for instance, showed strong upregulation of *zntA*, a gene encoding a P-type ATPase that functions as a zinc efflux pump. This gene plays a dual role in protecting the cell from toxic levels of zinc and potentially providing cross-resistance to other heavy metals, including silver [48]. Additionally, *copA*, a gene encoding a copper-transporting ATPase, was variably expressed among the mutants. In SAM7, *copA* was downregulated, which might indicate a shift in the cell’s strategy to manage copper and silver ion homeostasis more efficiently under stress.
3. Amino Acid Biosynthesis and Metabolism Genes: SAM3 and SAM6 exhibited notable upregulation in genes involved in amino acid biosynthesis and metabolism, such as *gltB* (glutamate synthase large subunit) and *asd* (aspartate-semialdehyde dehydrogenase). *GltB* plays a crucial role in the synthesis of glutamate, a key amino acid involved in nitrogen metabolism and as a precursor for other amino acids [49]. *Asd* is involved in the biosynthesis of lysine and other essential metabolites. The upregulation of these genes suggests an increased demand for amino acid synthesis, likely to support protein repair and synthesis during stress conditions. The prioritization of these metabolic pathways may help sustain cellular function and growth during silver exposure, providing a buffer against the detrimental effects of stress.
4. Cell Envelope Integrity Genes: Several mutants, particularly SAM7, showed upregulation of genes involved in maintaining cell envelope integrity. For instance, *murein* (peptidoglycan synthesis genes) such as *mrcA* (penicillin-binding protein 1A) and *lpoA* (lipoprotein involved in peptidoglycan synthesis) were upregulated. These genes are critical for maintaining the structural integrity of the bacterial cell wall, especially under conditions where membrane integrity might be compromised by silver ions.
5. Regulatory Functions Genes: The SAM mutants exhibited differential expression in several genes associated with regulatory functions. For example, *rpoA* (RNA polymerase alpha subunit) and *rpoS* (sigma factor S) were upregulated in SAM6, suggesting a heightened global stress response. *RpoS* is particularly important for the bacterial stress response, regulating the expression of numerous genes involved in survival during the stationary phase and under various environmental stresses. The upregulation of *rpoS* indicates that SAM6 might have an enhanced ability to manage multiple stressors, contributing to its superior fitness and resistance profile.
6. Metabolic Suppression Genes: Interestingly, several mutants, especially SAM7, exhibited downregulation of genes involved in central metabolism, including those associated with glycolysis (*pfkA*, phosphofructokinase) and the TCA cycle (*sdhA*, succinate dehydrogenase). This downregulation may reflect a strategic metabolic shift to conserve energy and resources, redirecting them toward essential stress responses and repair mechanisms. This metabolic suppression likely serves as a trade-off, allowing the cells to prioritize survival overgrowth in the presence of silver.

These widespread changes in gene expression across the SAM mutants highlight the complex and multifaceted nature of bacterial adaptation to silver stress. The upregulation of stress response genes, such as *msrP* and *huiH*, coupled with alterations in metal ion transport systems (*zntA*, *copA*), amino acid metabolism (*gltB*, *asd*), and regulatory networks (*rpoA*, *rpoS*), underscores the importance of coordinated genetic responses in developing robust resistance. The downregulation of metabolic genes, particularly in SAM7, suggests a strategic shift in cellular priorities, where survival mechanisms are favored over growth and energy production. 

The differential expression of genes involved in cell envelope integrity and global stress response further emphasizes the role of epistasis in fine-tuning these adaptive responses. By modifying multiple pathways simultaneously, the SAM mutants demonstrate the intricate interplay between genetic mutations and environmental pressures, leading to the emergence of distinct resistance phenotypes. This comprehensive adaptation strategy, involving both upregulation of protective mechanisms and downregulation of non-essential pathways, highlights the sophisticated nature of bacterial evolution in response to antimicrobial agents. 

### 3.3. Comparative Fitness Analysis 

**Figure 3:**
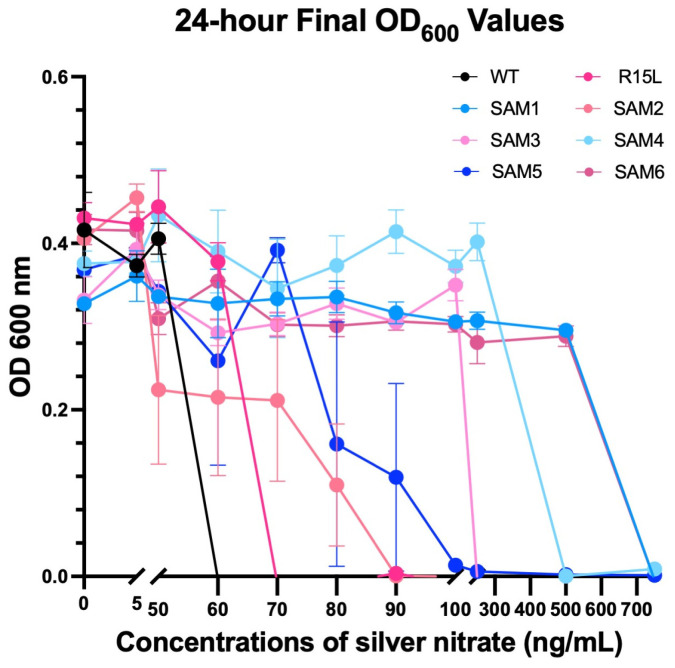
24-hour final OD600 values from growth response assays of *E. coli* populations to increasing silver nitrate concentrations. The 24-hour time point from growth response assays of WT, R15L, and SAM1-6 populations in Davis Minimal Broth (DMB) is shown under increasing concentrations of silver nitrate (0–750 ng/mL). OD600 values were measured hourly over 24 h, matching the selection time point used in the original experimental evolution study where the SAM populations evolved. Data were collected in triplicate, with means and standard errors of the mean (SEMs) plotted using GraphPad Prism. To calculate statistical variation between each time point and the WT, we performed a two-way ANOVA with multiple comparisons. Detailed statistical results are provided in Table S2. These growth curves were also used to determine the minimum inhibitory concentration (MIC), defined as the lowest silver concentration at which no growth was observed for a population.

**Figure 4:**
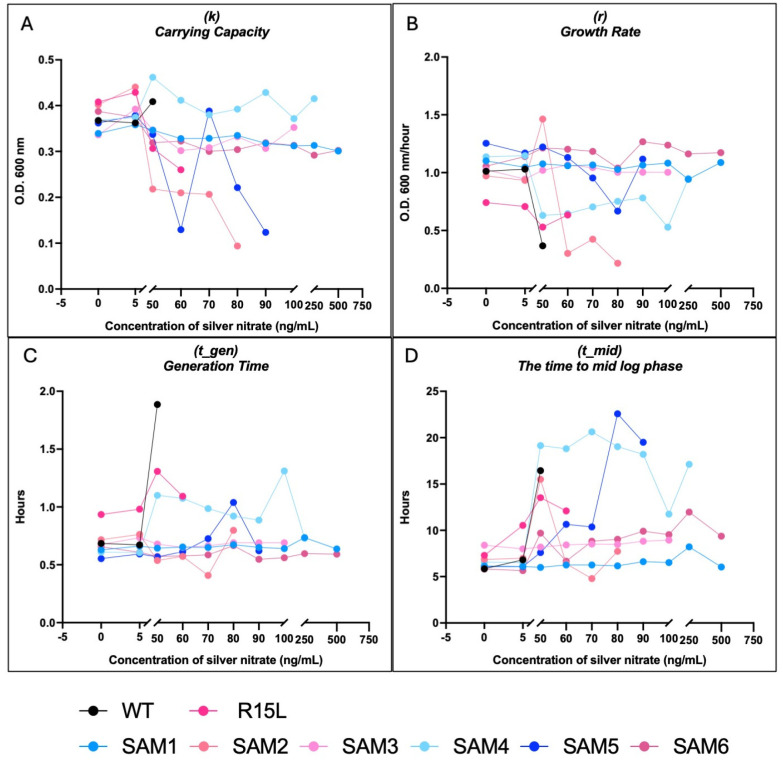
Detailed growth metrics across silver nitrate concentrations. This figure presents detailed growth metrics for each *E. coli* population under varying silver nitrate concentrations, analyzed using the R package Growthcurver (v0.3.1). Growthcurver fits the growth data to a logistic model, providing key metrics that describe the dynamics of each population. These metrics include growth rate (r), reflecting how quickly the population grows; generation time (t_gen), indicating the time required for the population to double; midpoint time (t_mid), representing the time at which the population reaches half its carrying capacity; and carrying capacity (k), denoting the maximum population size supported under the given conditions. The data generated by Growthcurver were plotted using GraphPad Prism. (**A**) (k) carrying capacity, (**B**) (r) on growth rate, (**C**) on generation time, and (**D**) on time to mid-log phase. These results, derived from Growthcurver’s comprehensive analysis, highlight the impact of silver stress on growth dynamics and reveal the differing adaptive responses among the populations.

**Figure 5:**
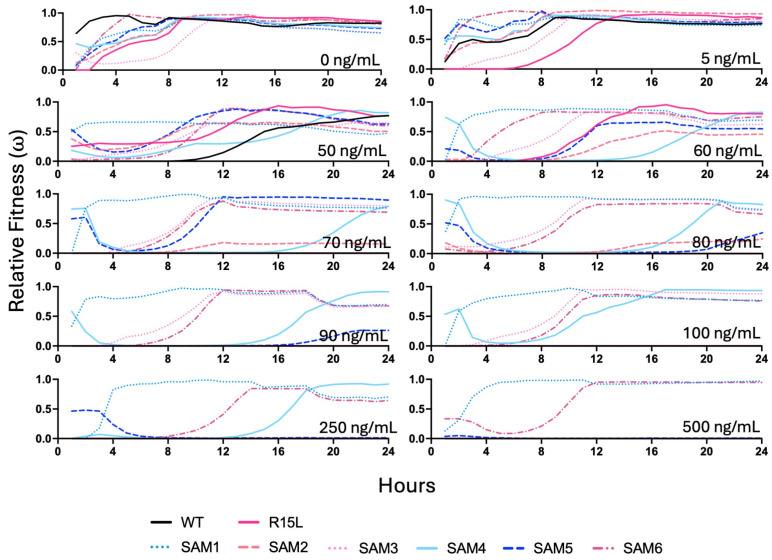
Comparative fitness of *E. coli* populations under silver nitrate stress. Relative fitness (ω) of each *E. coli* population under varying concentrations of silver nitrate (0–750 ng/mL) is shown. To calculate relative fitness, the OD_600_ of each population was divided by the maximum OD_600_ observed among other genotypes in the population at the same time points. Prior to this calculation, all negative growth values were set to 0 to ensure accurate comparisons, and the data were plotted in GraphPad Prism. Statistical analyses were conducted using a one-way ANOVA with pairwise multiple comparisons (Table S3) in GraphPad Prism. These results underscore the impact of silver stress on the competitive dynamics among the populations.

Growth assays were conducted over a range of silver nitrate concentrations (0–750 ng/mL) to evaluate the fitness and adaptability of the SAM mutants (Supplemental Figure S1 for full growth curves at all silver nitrate concentrations and Figure 3). The Growthcurver package was used to extract detailed growth metrics for each population, including the growth rate (r), generation time (t_gen), midpoint time (t_mid), and carrying capacity (k) (Figure 4). These metrics provided insights into how each strain adapts to varying levels of silver stress. Data from the 24-hour growth curves were also used to calculate relative fitness across silver concentrations and time points (Figure 5). It is important to note that relative fitness, as calculated in this study, reflects the performance of each population at a given time point in relation to the fittest population at that same time point, rather than indicating an absolute increase in fitness or resilience over time.

Growth curves showed that WT had an MIC of 60 ng/mL (Figure 3), which is consistent with previous studies that used a sub-MIC of 50 ng/mL silver nitrate for selection of silver resistance (37). At 50 ng/mL, WT displayed a drop in growth rate from 1 at 0 ng/mL to 0.36 at 50 ng/mL in addition to an increase from a time to mid-log from 6 h to 16 h, albeit while still maintaining its carrying capacity (Figure 4A–D).

The R15L single mutant has a slightly higher MIC of 70 ng/mL (Figure 3), albeit did not show much of a decline in growth rate across silver concentrations and only an increase from 7 to 12 h in its time to mid-log accounting for its increase in fitness over the WT at 50 ng/mL (Figure 5).

SAM1 consistently demonstrated the highest relative fitness (Figure 5) across most silver concentrations, with a growth rate (r) of 1.2 at 50 ng/mL and 1.15 at 750 ng/mL and stable carrying capacity (k), indicating strong adaptability and resistance, even under high silver stress (Figure 3 and Figure 4A,B). SAM6 also showed significant resilience, particularly at higher concentrations, with a slightly lower but still robust growth rate (r) of 1.18 at 50 ng/mL and 1.1 at 750 ng/mL. Both SAM1 and SAM6 had an MIC of 750 ng/mL, with short generation times (t_gen) and minimal lag phase extensions, highlighting their superior resistance (Figure 3 and Figure 4C). 

In contrast, WT and R15L-carrying populations, such as SAM2 and SAM3, struggled as silver concentration increased. SAM2 showed an MIC of 90 ng/mL. The growth rate dropped sharply from 1.0 at 50 ng/mL to 0.2 at 80 ng/mL, with a corresponding decline in carrying capacity and extended lag phases, suggesting reduced adaptability at higher concentrations. At lower silver concentrations, SAM2 exhibited statistically lower fitness than WT at 0 µg/mL, highlighting its inherent difficulty in thriving even without stress. SAM3, with an MIC of 250 ng/mL, showed steady metrics across concentrations of silver nitrate, including carrying capacity, growth rate, generation time, and time to mid-log phase.

SAM4, despite lacking a fixed *cusS* mutation, displayed strong resistance with an MIC of 500 ng/mL, though its growth rate and carrying capacity varied more significantly, reflecting a slower adaptive response. SAM5 showed moderate resistance with an MIC of 100 ng/mL, maintaining stable growth but struggling with higher silver stress. SAM4 and SAM5 both showed a large increase in their time to mid-log phase, at almost 20 h from SAM4 above silver concentrations of 50 ng/mL, and for SAM5 it remained around 10 h until 80 ng/mL and rose to 20 h at 90 ng/mL, where both are at ~6 h in absence of silver nitrate.

The differential fitness among the SAM mutants likely results from a variation in epistatic interactions between mutations. In particular, this is evident in the SAM populations that carry R15L mutations while all showing drastically different relative fitness levels, MIC, and growth metrics.

## 4. Discussion

The R15L *cusS* mutation has been predicted to be central to silver resistance through the upregulation of the *cus* efflux system [37]. However, as demonstrated in this study, its efficacy is not uniform across all silver-adapted populations. This variability can be attributed to the distinct genetic compositions of each population, which modulate how the *cusS* mutation interacts with other mutations in regulatory genes such as *ompR*, *rho*, and *fur* [5]. These interactions lead to different epistatic effects that either enhance or diminish the silver resistance phenotype in each population.

For instance, populations such as SAM6 and SAM7, which share the R15L mutation, exhibit superior resistance due to additional mutations in other regulatory genes, whereas populations lacking these additional mutations show lower resistance. This highlights that while the R15L *cusS* mutation plays a significant role, the broader genetic background is crucial in determining the overall efficacy of silver resistance.

Consequently, our findings suggest that the R15L *cusS* mutation alone is not universally applicable as a predictor of resistance across all bacterial strains. Instead, its impact is highly context-dependent, shaped by the specific genetic and environmental conditions of each population. This context dependency complicates the development of universal models of bacterial resistance and suggests that evolutionary trajectories may vary widely among populations, even when key adaptive mutations are shared.

### 4.1. Role of Epistasis in Silver Resistance

The variations in gene expression and fitness among the SAM mutants underscore the critical role of epistatic interactions in shaping bacterial adaptation to silver stress. The combination of mutations in *cusS*, *ompR*, *rho*, and other regulatory genes creates a more robust and adaptive response to silver stress than would be expected from single mutations alone.

Comparative analysis reveals that SAM mutants with *cusS* mutations, particularly those combined with *ompR* and *rho* mutations, generally exhibit a more integrated and potent resistance phenotype. This suggests a synergistic effect, where the combined impact of these mutations enhances the overall fitness and resistance of the bacterium beyond the additive effects of individual mutations suggesting positive epistasis. The epistatic interactions observed in SAM6, for instance, likely contribute to its exceptional resistance and fitness, as evidenced by its ability to maintain growth under high silver concentrations. In contrast, SAM1, which lacks a fixed *cusS* mutation, may rely on alternative epistatic interactions involving different regulatory pathways, resulting in a distinct but equally effective adaptive strategy. 

Our findings highlight that the adaptive advantage conferred by the *cusS* mutations is significantly modulated by their interaction with mutations in regulatory genes such as *ompR* and *rho*. These interactions do not merely enhance silver resistance but also reconfigure global stress response networks, leading to a more integrated and resilient phenotype. 

### 4.2. New Adaptive Traits That Are Not Predictable from the Individual Effects of Each Mutation Alone

We further assessed potential epistatic effects in these populations by identifying the commonly differentially expressed genes between the R15L strain and the SAM populations that carry the R15L mutation (SAM2, SAM3, SAM6, and SAM7). We then compared their differential gene expression profiles under conditions with and without silver exposure (Table 2). Specifically, in the presence of silver nitrate, the differential expression data strongly suggest that the enhanced silver resistance in SAM mutants is a result of positive epistasis, where the interaction between the R15L mutation in the *cusS* gene and additional mutations in the SAM strains leads to a phenotype that is greater than the sum of its parts.

This positive epistasis is evident in the opposing gene expression patterns observed between R15L and the SAM strains. For example, genes like *znuA* and *copA*, which are upregulated in the R15L strain, are downregulated in the SAM mutants. This suggests that the additional mutations in SAM strains do not simply add to the effects of the *cusS* mutation but instead modify the overall regulatory network, leading to a more effective response to silver. The SAM strains are not just following the same pathway as R15L but have developed alternative or enhanced pathways due to the combined effect of multiple mutations. 

The concept of positive epistasis is important here as it demonstrates how the interaction between mutations can lead to new adaptive traits that are not predictable from the individual effects of each mutation alone. In the case of the SAM mutants, the combined genetic changes result in a bacterium that is significantly more resistant to silver than the R15L strain, highlighting the power of epistatic interactions in driving evolutionary adaptation. This phenomenon explains why the SAM mutants, with their complex genetic backgrounds, exhibit such a robust resistance phenotype, far surpassing what would be expected from the *cusS* mutation in isolation. 

### 4.3. Mechanisms of Silver Resistance

The analysis of the SAM populations reveals distinct but overlapping mechanisms of silver resistance, each shaped by unique genetic modifications. While there is some clear overlap in these mechanisms, each population follows its own evolutionary trajectory, leading to variations in silver tolerance. These data have been summarized in Table 3.

SAM1 demonstrates the highest relative fitness and an MIC of 750 ng/mL, showing strong adaptability even under high silver stress. This resistance is achieved through a robust efflux system and a key mutation in *rpoC* that enhances transcriptional regulation. SAM1 adapts to silver stress by downregulating traditional metal detoxification pathways (*copA* and *cusF*) while upregulating alternative metal transporters like *zntA*, reflecting a complex and effective resistance strategy. 

SAM6 also exhibits significant resilience, with an MIC of 750 ng/mL. The combination of mutations in *cusS*, *fur*, *rpoA*, *ompR*, and *rho* supports metal ion efflux, transcriptional control, and outer membrane integrity. The R15L *cusS* mutation enhances the activity of the *cus* efflux system, while the upregulation of *msrQ* and *zinT* provides additional protection against oxidative stress and metal sequestration. SAM6’s broad resistance strategy includes the use of other efflux pumps like *emrD* and potential reductions in membrane permeability, ensuring efficient neutralization of silver ions. 

SAM2 exhibits moderate resistance (MIC of 90 ng/mL) but struggles at higher silver concentrations. It relies on upregulation of the *cus* operon, driven by the R15L mutation, to export toxic metal ions. A mutation in *dnaK* enhances protein damage management, while a deletion in *ompR* may reduce silver ion influx. The upregulation of *zntA* suggests potential cross-resistance to various metals, although SAM2’s overall fitness declines significantly at higher concentrations. 

SAM3 shares similar mechanisms with SAM2 but shows a broader resistance strategy through more pronounced upregulation of *zntA*. This contributes to its moderate resistance (MIC of 250 ng/mL), although SAM3 also struggles under high silver stress, with extended lag phases indicating difficulty in adaptation. 

SAM4 displays strong resistance (MIC of 500 ng/mL) despite lacking a fixed *cusS* mutation. Its unique resistance mechanisms center around RNA processing, nucleotide biosynthesis, and nitrogen metabolism. The mutations in *rph* and *pyrE* suggest a reprogramming of these processes, enhancing survival under stress. SAM4’s ability to adapt appears slower, with significant lag phases at high silver concentrations, potentially due to ongoing selection pressures. 

SAM5 shows moderate resistance (MIC of 100 ng/mL) and employs several core resistance mechanisms similar to SAM4. However, it is distinguished by a mutation in *cyaA*, which suggests alterations in cAMP signaling pathways, allowing SAM5 to finely regulate stress responses and metabolism. 

SAM7, while sharing many resistance mechanisms with SAM6, is characterized by mutations near transposable elements, providing greater genetic flexibility and adaptability. This could allow SAM7 to dynamically alter gene expression in response to environmental changes, offering a potential advantage in fluctuating environments. 

These findings underscore the role of epistasis and genetic background in shaping the resistance strategies of each SAM population. While the R15L *cusS* mutation is central to the resistance mechanisms observed, its effectiveness varies depending on the presence of additional mutations that modulate stress responses and metabolic processes. 

### 4.4. Crosstalk and Adaptation in TCRS: Evaluating a Three-Step Model 

In our previous work, we proposed a three-step model for how adaptive mutations in TCRS genes drive bacterial adaptation, focusing on genotype-environment interactions (38). Initially based on single mutations in the *cusS* TCRS gene, to this model we can now add four SAM mutants (SAM2, SAM3, SAM6, and SAM7) that carry fixed mutations in *cusS* but with distinct genetic backgrounds due to additional mutations, allowing us to include the role of epistasis in the model. 

Primary Response: The first step in our model involves the upregulation of TCRS genes with adaptive mutations, leading to constitutive expression of response genes, even without stimuli. As with the single adaptive mutants such as R15L, SAM mutants carrying *cusS* mutations (SAM2, SAM3, SAM6, and SAM7) also exhibit upregulation and constitutive expression of the *cus* genes, aligning with our previous findings. However, differences in *cus* gene expression suggest varying resistance profiles. SAM6 shows the highest *cus* expression, correlating with its superior resistance (MIC of 750 ng/mL), suggesting additional mutations amplify its efflux capacity. SAM7, similar to SAM6, may benefit from genetic flexibility due to transposable elements, aiding its adaptation to environmental changes. 

Epistasis plays a crucial role in shaping these outcomes. For example, while SAM2 and SAM3 share similar mechanisms, their different genetic backgrounds lead to variations in fitness and expression levels. SAM6, with its combination of mutations, exhibits the most refined response, highlighting how epistasis can enhance or modify the effectiveness of resistance genes like those in the *cus* operon. 

Secondary Response: The second step in the model involves the differential expression of CusR-regulated genes, often through cross-talk with other TCRS, broadening the cell’s adaptive response. SAM mutants with *cusS* mutations show differential expression in CusR regulated genes, such as a significant downregulation of *cueO* and *copA*, though this varies among the mutants. SAM2 and SAM3 exhibit strong downregulation of *cueR*, *phoP*, and *rpoS*, amplifying the repression of these genes. SAM6, however, does not downregulate *cueO* or *copA*, possibly due to epistatic interactions that redirect its secondary response to other pathways, such as osmotic stress. 

Cross-talk between CusR and other systems like HprR/HprS also illustrates the complexity of this response [50] SAM populations with the R15L mutation show varied upregulation of redox and oxidative stress-related genes, with differences in expression indicating that epistasis and genetic background are modifying how each strain manages cross-talk. Additionally, *ompR* mutations in some SAM populations further modify this response, affecting outer membrane integrity and stress responses. SAM6 and SAM7, for instance, focus on osmotic stress and metal ion toxicity while deprioritizing other stress responses. 

Tertiary Response: The final step in the model involves the “fitness tuning” through additional gene expression changes that optimize the cell’s fitness in its original environment. SAM2, SAM3, SAM6, and SAM7 show upregulation in metal homeostasis, indicating a strong adaptation to resist metal toxicity, with SAM7 exhibiting the highest upregulation. Metabolic activity is generally downregulated to conserve energy under stress, with SAM7 showing the most significant suppression. Transport system activity varies, with SAM2 showing strong upregulation while SAM6 exhibits substantial downregulation, suggesting different adaptive strategies. SAM3 and SAM6 prioritize amino acid biosynthesis and metabolism, while SAM2 and SAM7 focus on other responses like metal homeostasis. Motility-related genes are moderately upregulated in SAM3, SAM6, and SAM2, indicating an adaptive strategy involving increased motility. The differential expression across these mutants underscores the role of epistasis and genetic background in shaping their tertiary responses, leading to different survival strategies. 

These findings illustrate the complexity of bacterial adaptation, where cross-talk between regulatory systems and epistatic interactions create finely tuned survival mechanisms. The R15L *cusS* mutation primarily confers silver resistance through the upregulation of the *cus* efflux system, but its effectiveness varies among SAM populations due to their unique genetic backgrounds. This adaptability is crucial for these mutants to thrive under diverse environmental conditions. 

### 4.5. Refinements and Modifications to the Model 

**Figure 6:**
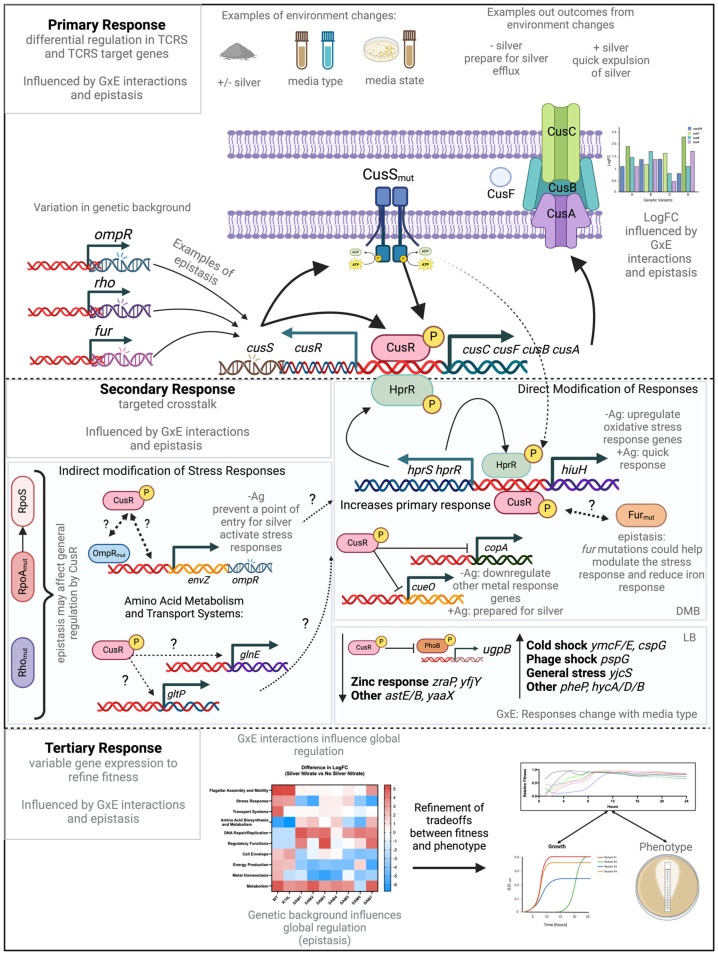
Refined model of adaptive responses in silver-resistant *E. coli* Mutants: A refined three-step adaptive response mechanism in silver-resistant *E. coli* mutants is illustrated, emphasizing the roles of epistasis and genotype-by-environment (GxE) dynamics throughout the adaptive process. Dotted arrows indicate interactions that have not yet been characterized, while thick, bold arrows represent the most critical pathways in the response. The model begins with the Primary Response, where epistatic interactions among mutations in the *cusS* gene and other regulatory genes lead to the enhanced expression of the CusSR two-component regulatory system (TCRS) and its downstream efflux pump genes, *cusCFBA*. These interactions, influenced by specific environmental conditions, establish a baseline level of silver resistance that varies across SAM populations due to GxE dynamics. The combined effects of these mutations create a context-dependent expression of resistance traits.

Our findings provide key insights that refine our existing model of bacterial adaptation, particularly in the context of genotype-by-environment (GxE) interactions. These refinements are directly informed by the new data (Figure 6).

#### 4.5.1. Epistasis in GxE Interactions 

Findings: The varying gene expression and fitness profiles among the SAM mutants, especially those with the R15L *cusS* mutation, underscore the critical role of epistatic interactions. For instance, SAM6, with additional mutations in *ompR*, *rho*, and *fur*, exhibits enhanced resistance compared to SAM2 and SAM3, despite sharing the same *cusS* mutation. 

In the Secondary Response, epistasis and GxE interactions further shape the crosstalk between CusR and other regulatory pathways, such as those governing oxidative stress and osmotic stress responses. These pathways are differentially activated depending on the specific genetic background and environmental conditions of each mutant. For instance, SAM6, carrying additional mutations in ompR, rho, and fur, exhibits heightened resistance through enhanced modulation of these secondary pathways. Conversely, SAM2 and SAM3, with their unique epistatic backgrounds, show varied pathway activation and resistance levels. 

The Tertiary Response, or “fitness tuning”, involves the fine-tuning of gene expression to optimize survival and resistance under the specific environmental conditions experienced by each population. This stage is driven by epistatic interactions that shape unique expression patterns, maximizing fitness in a context-specific manner. GxE interactions further refine these adaptations, with populations like SAM6 and SAM7 displaying distinct adaptive strategies. SAM7, for example, exhibits greater adaptability due to mutations near transposable elements, while SAM4 relies on shifts in RNA processing and metabolism to survive in silver-rich environments. This refined model underscores the integral role of epistasis and GxE interactions in shaping the evolutionary trajectories and adaptive changes driven by mutations in TCRS genes, highlighting why these genes are often selected for adaptation. This figure was created using BioRender.

Model Refinement: Epistasis is now emphasized as a key factor in GxE interactions, shaping how genetic changes influence adaptive traits across different environments. The superior resistance in SAM6 results from these interactions, demonstrating that adaptation is driven by both genetic interactions and environmental pressures. 

#### 4.5.2. Impact of Genetic Background 

Findings: The study highlights the influence of genetic background on adaptation. SAM7, despite sharing key mutations with SAM6, shows greater adaptability due to mutations near transposable elements. 

Model Refinement: The model now reinforces the idea that genetic background plays a crucial role in GxE interactions. Mutations in regulatory elements, such as those found in SAM6, can significantly alter adaptive outcomes under varying environmental conditions, further illustrating the context-dependent nature of bacterial adaptation. 

#### 4.5.3. Multifunctional Pathways in GxE Dynamics 

Findings: Adaptive responses in SAM mutants involve not only metal ion efflux but also broader pathways like oxidative stress response and RNA processing. SAM4 compensates for the lack of a fixed *cusS* mutation by upregulating RNA processing genes, aiding in survival under silver stress. 

#### 4.5.4. Adaptive Strategy Prioritization 

Findings: Different SAM populations prioritize specific adaptive strategies depending on their genetic makeup. For instance, SAM6 focuses on metal ion efflux, while SAM4 emphasizes RNA processing and metabolic shifts. 

Model Refinement: The revised model recognizes that bacterial populations adopt varied adaptive strategies within the GxE framework. Adaptation is not uniform but involves the selective prioritization of pathways that best address the environmental challenges faced by each population.

### 4.6. Revised Model Implications

In light of these findings, our revised model now emphasizes the critical role of epistasis in not only amplifying resistance traits but also in reprogramming broader regulatory networks that enable the bacteria to thrive under varying environmental stresses. This revised perspective suggests that the evolutionary pathways leading to robust resistance are more complex and involve a greater degree of interaction between multiple genetic and environmental factors than previously understood.

## 5. Conclusions

This study underscores the complexity of bacterial resistance mechanisms, particularly in the context of evolving resistance to silver. Our findings reveal that identifying a single mutation associated with resistance, such as the R15L mutation, does not guarantee that the organism will exhibit resistance. The role of genetic background is critical, as evidenced by the dramatically different resistance profiles among populations carrying the same R15L mutation. Moreover, SAM1, which does not harbor any distinctive silver resistance mutations yet demonstrates the greatest fitness and highest resistance, highlights the intricate interplay of genetic background and epistatic interactions. 

These insights have significant clinical implications. As sequencing technology becomes more affordable and widely used for evaluating antibiotic resistance, it is crucial to consider the broader genetic context rather than relying solely on single-gene assessments. The variability in resistance profiles among the SAM populations challenges the predictive power of identifying single mutations and suggests that bacterial adaptation is driven by a dynamic network of genetic interactions that extend beyond individual mutations. 

The unique resistance strategies observed across the SAM mutants exemplify how bacteria harness multiple interacting mutations to reshape cellular processes, leading to more robust and adaptable phenotypes. This adaptability not only allows bacteria to survive in hostile environments but also to exploit new ecological niches, emphasizing the importance of genetic diversity and the potential for rapid evolution in microbial populations. 

In a broader evolutionary context, these findings highlight the potential for rapid and complex evolution in response to strong selective pressures, such as antimicrobial agents. This suggests that strategies targeting single pathways may be insufficient to curb the development of resistance. Instead, a more comprehensive approach, considering epistasis and the broader genetic landscape, is necessary to combat bacterial resistance effectively.

The evolutionary trajectories observed in the SAM mutants provide valuable insights into the dynamic and multifaceted nature of microbial adaptation. These insights contribute to a deeper understanding of how bacteria evolve complex resistance mechanisms and underscore the need for innovative strategies to address the growing challenge of antimicrobial resistance in both clinical and environmental settings.

## Figures and Tables

**Table 1:** BreSeq-detected moderate to high-frequency mutations (>0.25) in SAM populations following DNA sequencing: mutations highlighted in the table were detected to fixation.

	Location	Mutation	Frequency	Annotation	Gene	Description
SAM1	847,955	G→A	1.000	A17V (GCG→GTG)	*glnH* ←	glutamine transporter subunit
	4,188,510	C→A	0.877	T1054N (ACC→AAC)	*rpoC* →	RNA polymerase, beta prime subunit
	1,868,984	C→A	0.849	N10K (AAC→AAA)	*yeaH* →	UPF0229 family protein
	3,815,809	Δ1 bp	0.807	intergenic (−41/+25)	*pyrE* ←/← *rph*	orotate phosphoribosyltransferase/ribonuclease PH (defective);enzyme; Degradation of RNA; RNase PH
	3,831,168	C→T	0.732	L238L (CTA→TTA)	*yicH* →	putative inner membrane-anchored periplasmic AsmA family protein
SAM2	12,661	C→G	1.000	R167G (CGT→GGT)	*dnaK* →	chaperone Hsp70, with co-chaperone DnaJ
	594,727	C→A	1.000	R15L (CGC→CTC)	*cusS* ←	sensory histidine kinase in two-component regulatory system with CusR, senses copper ions
	3,101,306	G→T	1.000	I106I (ATC→ATA)	*yggN* ←	DUF2884 family putative periplasmic protein
	3,133,461	G→A	1.000	A166V (GCA→GTA)	*yghS* ←	putative ATP-binding protein
	3,536,061	Δ1 bp	1.000	coding (524/720 nt)	*ompR* ←	response regulator in two-component regulatory system with EnvZ
	3,816,605	Δ103 bp	1.000		*[yicC]*	*[yicC]*
	3,843,548	A→C	1.000	I81R (ATA→AGA)	*yicO* ←	putative adenine permease
	3,966,612	C→T	1.000	R66C (CGT→TGT)	*rho* →	transcription termination factor
	3,992,735	C→A	0.930	S528 * (TCG→TAG)	*cyaA* →	adenylate cyclase
	3,177,973	IS*1* (+) +9 bp	0.552	intergenic (−63/−134)	*nudF* ←/→ *tolC*	ADP-ribose pyrophosphatase/transport channel
	915,226	T→C	0.446	intergenic (−369/+126)	*ybjE* ←/← *aqpZ*	putative transporter/aquaporin Z
	2,229,177	A→G	0.376	intergenic (+112/+261)	*yohP* →/← *dusC*	uncharacterized protein/tRNA-dihydrouridine synthase C
	4,296,060	C→T	0.282	intergenic (+266/+376)	*gltP* →/← *yjcO*	glutamate/aspartate:proton symporter/Sel1 family TPR-like repeat protein
SAM3	12,661	C→G	1.000	R167G (CGT→GGT)	*dnaK* →	chaperone Hsp70, with co-chaperone DnaJ
	594,727	C→A	1.000	R15L (CGC→CTC)	*cusS* ←	sensory histidine kinase in two-component regulatory system with CusR, senses copper ions
	3,101,306	G→T	1.000	I106I (ATC→ATA)	*yggN* ←	DUF2884 family putative periplasmic protein
	3,133,461	G→A	1.000	A166V (GCA→GTA)	*yghS* ←	putative ATP-binding protein
	3,536,061	Δ1 bp	1.000	coding (524/720 nt)	*ompR* ←	response regulator in two-component regulatory system with EnvZ
	3,816,605	Δ103 bp	1.000		*[yicC]*	*[yicC]*
	3,843,548	A→C	1.000	I81R (ATA→AGA)	*yicO* ←	putative adenine permease
	3,966,612	C→T	1.000	R66C (CGT→TGT)	*rho* →	transcription termination factor
	3,992,735	C→A	0.865	S528 * (TCG→TAG)	*cyaA* →	adenylate cyclase
	3,177,973	IS*1* (+) +9 bp	0.648	intergenic (−63/−134)	*nudF* ←/→ *tolC*	ADP-ribose pyrophosphatase/transport channel
SAM4	3,992,735	C→A	0.865	S528 * (TCG→TAG)	*cyaA* →	adenylate cyclase
	3,177,973	IS*1* (+) +9 bp	0.648	intergenic (−63/−134)	*nudF* ←/→ *tolC*	ADP-ribose pyrophosphatase/transport channel
	3,360,120	G→A	0.163	R308H (CGC→CAC)	*gltD* →	glutamate synthase, 4Fe-4S protein, small subunit
	4,296,060	C→T	0.159	intergenic (+266/+376)	*gltP* →/← *yjcO*	glutamate/aspartate:proton symporter/Sel1 family TPR-like repeat protein
	2,725,169	Δ1 bp	0.147	coding (578/1299 nt)	*kgtP* ←	alpha-ketoglutarate transporter
	2,229,205	G→C	0.116	intergenic (+140/+233)	*yohP* →/← *dusC*	uncharacterized protein/tRNA-dihydrouridine synthase C
	3,815,859	Δ82 bp	0.533		*[rph]*–*[rph]*	*[rph]*, *[rph]*
	3,815,824	G→T	0.277	intergenic (−56/+10)	*pyrE* ←/← *rph*	orotate phosphoribosyltransferase/ribonuclease PH (defective);enzyme; Degradation of RNA; RNase PH
	3,198,033	IS*186* (+) +6 bp :: Δ2 bp	0.258	coding (1604–1609/2841 nt)	*glnE* ←	fused deadenylyltransferase/adenylyltransferase for glutamine synthetase
	4,296,060	C→T	0.235	intergenic (+266/+376)	*gltP* →/← *yjcO*	glutamate/aspartate:proton symporter/Sel1 family TPR-like repeat protein
	3,815,809	Δ1 bp	0.138	intergenic (−41/+25)	*pyrE* ←/← *rph*	orotate phosphoribosyltransferase/ribonuclease PH (defective);enzyme; Degradation of RNA; RNase PH
	915,226	T→C	0.112	intergenic (−369/+126)	*ybjE* ←/← *aqpZ*	putative transporter/aquaporin Z
	594,727	C→A	0.055	R15L (CGC→CTC)	*cusS* ←	sensory histidine kinase in two-component regulatory system with CusR, senses copper ions
SAM5	3,197,294	T→A	0.408	E783V (GAA→GTA)	*glnE* ←	fused deadenylyltransferase/adenylyltransferase for glutamine synthetase
	3,815,809	Δ1 bp	0.373	intergenic (−41/+25)	*pyrE* ←/← *rph*	orotate phosphoribosyltransferase/ribonuclease PH (defective);enzyme; Degradation of RNA; RNase PH
	3,815,859	Δ82 bp	0.367		*[rph]*–*[rph]*	*[rph]*, *[rph]*
	4,296,060	C→T	0.236	intergenic (+266/+376)	*gltP* →/← *yjcO*	glutamate/aspartate:proton symporter/Sel1 family TPR-like repeat protein
	3,992,588	(ATCAGCC)_2→1_	0.235	coding (1436–1442/2547 nt)	*cyaA* →	adenylate cyclase
	1,907,503	IS*3* (–) +5 bp :: +T	0.178	coding (85–89/144 nt)	*yobF* ←	DUF2527 family heat-induced protein
	4,181,669	A→G	0.164	E142G (GAG→GGG)	*rpoB* →	RNA polymerase, beta subunit
	3,485,966	IS*2* (+) +5 bp	0.129	intergenic (−148/−150)	*yhfA* ←/→ *crp*	OsmC family protein/cAMP-activated global transcription factor, mediator of catabolite repression
	3,897,059	C→A	0.129	L96I (CTC→ATC)	*yieH* →	phosphoenolpyruvate and 6-phosphogluconate phosphatase
SAM6	594,727	C→A	1.000	R15L (CGC→CTC)	*cusS* ←	sensory histidine kinase in two-component regulatory system with CusR, senses copper ions
	710,620	C→A	1.000	K9N (AAG→AAT)	*fur* ←	ferric iron uptake regulon transcriptional repressor; autorepressor
	3,440,186	C→A	1.000	V282L (GTA→TTA)	*rpoA* ←	RNA polymerase, alpha subunit
	3,536,041	G→A	1.000	R182C (CGC→TGC)	*ompR* ←	response regulator in two-component regulatory system with EnvZ
	3,966,604	G→T	1.000	G63V (GGT→GTT)	*rho* →	transcription termination factor
	4,232,641	C→A	0.318	R198L (CGT→CTT)	*lysC* ←	lysine-sensitive aspartokinase 3
	1,213,820	G→C	0.279	D80E (GAC→GAG)	*bluR* ←	repressor of blue light-responsive genes
	3,359,461	Δ1 bp	0.226	coding (264/1419 nt)	*gltD* →	glutamate synthase, 4Fe-4S protein, small subunit
SAM7	594,727	C→A	1.000	R15L (CGC→CTC)	*cusS* ←	sensory histidine kinase in two-component regulatory system with CusR, senses copper ions
	710,620	C→A	1.000	K9N (AAG→AAT)	*fur* ←	ferric iron uptake regulon transcriptional repressor; autorepressor
	1,428,765	T→C	1.000	intergenic (−39/−30)	*insH1* ←/→ *lomR*	IS5 transposase and trans-activator; IS, phage, Tn; Transposon-related functions; extrachromosomal; transposon related/pseudogene, Rac prophage lom homolog; Phage or Prophage Related; interrupted by IS5 and N-ter deletion
	3,440,186	C→A	1.000	V282L (GTA→TTA)	*rpoA* ←	RNA polymerase, alpha subunit
	3,536,041	G→A	1.000	R182C (CGC→TGC)	*ompR* ←	response regulator in two-component regulatory system with EnvZ
	3,815,801	Δ1 bp	1.000	intergenic (−33/+33)	*pyrE* ←/← *rph*	orotate phosphoribosyltransferase/ribonuclease PH (defective);enzyme; Degradation of RNA; RNase PH
	3,966,604	G→T	1.000	G63V (GGT→GTT)	*rho* →	transcription termination factor
	1,212,080:1	+C	1.000	intergenic (−77/+623)	*iraM* ←/← *ycgX*	RpoS stabilizer during Mg starvation, anti-RssB factor/DUF1398 family protein
	1,213,820	G→C	0.627	D80E (GAC→GAG)	*bluR* ←	repressor of blue light-responsive genes
	3,359,461	Δ1 bp	0.590	coding (264/1419 nt)	*gltD* →	glutamate synthase, 4Fe-4S protein, small subunit
	4,296,060	C→T	0.248	intergenic (+266/+376)	*gltP* →/← *yjcO*	glutamate/aspartate:proton symporter/Sel1 family TPR-like repeat protein

* Indicates that the mutation resulted in the incorporation of a stop codon in place of the original amino acid.

**Table 2:** Evidence of epistasis: The table lists biological function and genes that are differentially regulated in R15L and all R15L-carrying SAM populations (SAM2, SAM3, SAM6, and SAM7) under both the absence and presence of silver nitrate.

In DMB Alone											
Biological Function	Gene	R15L_logFC		SAM2_logFC		SAM3_logFC		SAM6_logFC		SAM7_logFC	
Metal Homeostasis (Zinc Transport)	*znuA*	1.694	Up	−5.107	Down	−4.516	Down	−3.553	Down	−4.875	Down
Metal Homeostasis (Copper Export)	*copA*	−1.723	Down	−7.115	Down	−6.099	Down	−6.739	Down	−6.873	Down
Regulatory Functions (Redox Stress Response)	*hprR*	3.078	Up	2.780	Up	4.052	Up	1.609	Up	3.787	Up
Metal Homeostasis (Copper Detoxification)	*cueO*	−1.560	Down	−5.212	Down	−4.669	Down	−6.070	Down	−4.945	Down
Membrane Proteins (Potential Stress Response or Transport)	*shoB*	1.941	Up	3.827	Up	3.525	Up	4.512	Up	3.934	Up
Energy Production (Anaerobic Respiration)	*napH*	2.023	Up	4.341	Up	4.789	Up	4.199	Up	4.403	Up
Bacteriophage Interaction (Phage Entry)	*nfrB*	1.617	Up	2.174	Up	1.619	Up	2.803	Up	1.790	Up
Cell Envelope (Peptidoglycan Remodeling)	*mepM*	1.372	Up	−3.552	Down	−3.977	Down	−3.465	Down	−4.218	Down
Metal Homeostasis (Copper/Silver Efflux)	*cusA*	3.285	Up	3.167	Up	2.753	Up	3.237	Up	4.018	Up
Metal Homeostasis (Iron Transport)	*yfhH*	1.594	Up	2.597	Up	2.571	Up	3.292	Up	2.572	Up
Metal Homeostasis (Copper/Silver Efflux)	*cusB*	3.746	Up	3.164	Up	2.676	Up	3.026	Up	4.154	Up
Transport Systems (Amino Acid Transport)	*pheP*	2.550	Up	3.148	Up	2.715	Up	3.146	Up	3.517	Up
Nucleotide Metabolism (Purine Salvage Pathway)	*ghxP*	2.450	Up	5.917	Up	3.650	Up	2.340	Up	5.340	Up
**In Presence of 50 ng/mL Silver Nitrate**											
**Biological Function**	**Gene**	**R15L_logFC**		**SAM2_logFC**		**SAM3_logFC**		**SAM6_logFC**		**SAM7_logFC**	
Metal Homeostasis (Zinc Transport)	*znuA*	5.344	Up	−4.877	Down	−4.602	Down	−4.134	Down	−5.168	Down
Metal Homeostasis (Copper Export)	*copA*	5.769	Up	−6.581	Down	−6.517	Down	−5.676	Down	−7.027	Down
Regulatory Functions (Redox Stress Response)	*hprR*	−2.073	Down	2.971	Up	3.221	Up	2.544	Up	3.570	Up
Metal Homeostasis (Copper Detoxification)	*cueO*	5.807	Up	−5.137	Down	−4.975	Down	−5.726	Down	−5.079	Down
Membrane Proteins (Potential Stress Response or Transport)	*shoB*	−1.593	Down	3.410	Up	3.196	Up	3.972	Up	3.797	Up
Energy Production (Anaerobic Respiration)	*napH*	−3.244	Down	4.158	Up	4.843	Up	2.773	Up	4.864	Up
Bacteriophage Interaction (Phage Entry)	*nfrB*	−2.437	Down	2.269	Up	1.867	Up	3.743	Up	2.115	Up
Cell Envelope (Peptidoglycan Remodeling)	*mepM*	2.915	Up	−4.029	Down	−3.910	Down	−3.861	Down	−4.885	Down
Metal Homeostasis (Copper/Silver Efflux)	*cusA*	2.293	Up	3.184	Up	2.735	Up	3.129	Up	3.674	Up
Metal Homeostasis (Iron Transport)	*yfhH*	−2.223	Down	2.936	Up	2.577	Up	4.065	Up	2.348	Up
Metal Homeostasis (Copper/Silver Efflux)	*cusB*	2.827	Up	3.013	Up	2.097	Up	3.055	Up	3.690	Up
Transport Systems (Amino Acid Transport)	*pheP*	2.377	Up	2.968	Up	2.503	Up	2.918	Up	2.879	Up
Nucleotide Metabolism (Purine Salvage Pathway)	*ghxP*	−1.647	Down	5.197	Up	4.370	Up	3.033	Up	4.724	Up

Green-shaded rows indicate genes that are oppositely regulated between R15L and the SAM populations.

**Table 3:** Summary of growth metrics, genetic mutations, and mechanisms of silver resistance in silver-adapted *E. coli* populations.

Population	MIC (ng/mL)	Growth Metrics	Notable Mutations	Differential Expression	Mechanism of Silver Resistance	Notable Observations
SAM1	750	r: 1.2 (50 ng/mL), 1.15 (750 ng/mL); t_gen: Short; t_mid: Stable; k: Strong resistance	*glnH*, *rpoC*	Moderate upregulation of *zntA* and metal transporters, downregulation of *cus* efflux system	Alternative metal transport, downregulates *cus* system	Highest relative fitness, strong adaptability, short lag phase
SAM2	90	r: 1.0 (50 ng/mL), 0.2 (80 ng/mL); t_gen: Extended; t_mid: Lower fitness; k: Declines at high concentrations	*cusS*, *rho*, *ompR*	Downregulation of *cus* efflux genes, upregulation of *zntA* and *dnaK*	Moderate *cus* efflux activity, zinc efflux upregulation	Struggles at higher silver concentrations, fitness lower than WT at low silver
SAM3	250	r: Steady; t_gen: Steady; t_mid: Moderate; k: Varies	*cusS*, *rho*, *ompR*	Upregulation of *zntA*, moderate *cus* efflux expression	Balanced *cus* efflux and metal transport systems	Moderate resistance, extended lag phases under high silver stress
SAM4	500	r: Slower growth; t_gen: Significant increase; t_mid: ~20 h (above 50 ng/mL); k: Moderate resistance	*glnE*, *rph*	No *cus* efflux upregulation, upregulation of RNA processing genes	RNA processing, alternative metabolic adaptations	Adapts slowly, extended lag phase, lacks fixed *cusS* mutation
SAM5	100	r: Stable; t_gen: ~10 h (80 ng/mL), ~20 h (90 ng/mL); t_mid: ~10–20 h; k: Moderate resistance	*rph*, *glnE*	Downregulation of central metabolism, upregulation of *zntA*	Metabolic adjustments, zinc efflux	Moderate fitness, struggles with adaptation under higher silver stress
SAM6	750	r: 1.18 (50 ng/mL), 1.1 (750 ng/mL); t_gen: Short; t_mid: Fast; k: High carrying capacity	*cusS*, *fur*, *rpoA*, *ompR*, *rho*	Strong upregulation of *cus* efflux genes, *msrQ* and *zinT* for stress protection	High-efficiency *cus* efflux, stress defense upregulation	Superior resistance, robust response to high silver concentrations, short lag phase
SAM7	750	r: 1.15 (50 ng/mL), 1.1 (750 ng/mL); t_gen: Short; t_mid: Fast; k: High carrying capacity	*cusS*, *fur*, *rpoA*, *ompR*, *rho*	Highest upregulation of *cus* efflux genes, *rho* and *ompR* for membrane stability	Robust *cus* efflux, membrane integrity	Similar to SAM6, strong genetic flexibility due to transposable elements

## Data Availability

Public access code for the entire Bioproject is accessible through PRJNA1160277 and the individual datasets are also accessible directly through the SRA database using SAMN43760522-SAMN43760542.

## Supplementary Materials

The following supporting information can be downloaded at: https://www.mdpi.com/article/10.3390/microorganisms12102000/s1, Figure S1: Complete 24-hour growth response assays of *E. coli* populations to increasing silver nitrate concentrations. Table S1: All detected mutations from DNA sequencing analysis of all SAM populations. Table S2: Two-way ANOVA with pairwise comparisons of 24-hour growth data. Table S3: One-way ANOVA with multiple pairwise comparisons of relative fitness data.
