# Supplementary material for: It Takes Two to Make a Thing Go Right: Epistasis, Two-Component Response Systems, and Bacterial Adaptation
Source: Microorganisms. 2024 Sep 30;12(10):2000. doi: 10.3390/microorganisms12102000 (PMC11510482; doi:10.3390/microorganisms12102000)
Supplement: Supplementary file 1 [file microorganisms-12-02000-s001.zip › TableS1.pdf]

Table S1 - All detected mutations from DNA sequencing analysis of all SAM populations: Mutations detected a frequency below 0.1 are not highlighted.

	Location	Mutation	frequency	Annotation	Gene
	847,955	G→A	1.000	A17V (GCG→GTG)	*glnH* ←
	4,188,510	C→A	0.877	T1054N (ACC→AAC)	*rpoC* →
	1,868,984	C→A	0.849	N10K (AAC→AAA)	*yeaH* →
	3,815,809	Δ1 bp	0.807	intergenic (-41/+25)	*pyrE* ← / ← *rph*
	3,831,168	C→T	0.732	L238L (CTA→TTA)	*yicH* →
	4,296,060	C→T	0.178	intergenic (+266/+376)	*gltP* → / ← *yjcO*
	3,047,290	A→G	0.153	R584R (CGT→CGC)	*gcvP* ←
	294,161	IS*2* (+) +5 bp	0.079	coding (635-639/855 nt)	*yagM* ←
	2,067,300	C→T	0.075	G8R (GGA→AGA)	*insH1* ←
	1,899,974	G→A	0.065	P516P (CCG→CCA)	*yoaD* →
	1,717,681	C→A	0.062	E109D (GAG→GAT)	*pdxH* ←
	2,527,378	C→A	0.058	P145T (CCA→ACA)	*yfeH* →
	375,045	G→A	0.057	intergenic (+164/-414)	*mhpE* → / → *mhpT*
	1,466,639	C→T	0.057	pseudogene (1248/2513 nt)	*ydbA* →
	1,088,656	A→G	0.055	M401T (ATG→ACG)	*pgaB* ←
	3,264,853	C→T	0.051	M55I (ATG→ATA)	*tdcC* ←
SAM1	2,818,046	A→G	0.050	intergenic (-186/+13)	*argQ* ← / ← *argZ*
	12,661	C→G	1.000	R167G (CGT→GGT)	*dnaK* →
	594,727	C→A	1.000	R15L (CGC→CTC)	*cusS* ←
	3,101,306	G→T	1.000	I106I (ATC→ATA)	*yggN* ←
	3,133,461	G→A	1.000	A166V (GCA→GTA)	*yghS* ←
	3,536,061	Δ1 bp	1.000	coding (524/720 nt)	*ompR* ←
	3,816,605	Δ103 bp	1.000		*[yicC]*
	3,843,548	A→C	1.000	I81R (ATA→AGA)	*yicO* ←
	3,966,612	C→T	1.000	R66C (CGT→TGT)	*rho* →
	3,992,735	C→A	0.930	S528* (TCG→TAG)	*cyaA* →
	3,177,973	IS*1* (+) +9 bp	0.552	intergenic (-63/-134)	*nudF* ← / → *tolC*
	915,226	T→C	0.446	intergenic (-369/+126)	*ybjE* ← / ← *aqpZ*
	2,229,177	A→G	0.376	intergenic (+112/+261)	*yohP* → / ← *dusC*
	4,296,060	C→T	0.282	intergenic (+266/+376)	*gltP* → / ← *yjcO*
	118,951	C→T	0.089	S73S (TCC→TCT)	*ampD* →
	881,776	T→C	0.088	V350V (GTT→GTC)	*dacC* →
	1,195,125	G→A	0.082	M1M (ATG→ATA) †	*icd* →
	1,441,574	C→A	0.077	G57V (GGT→GTT)	*hslJ* ←
	1,829,888	G→A	0.064	A293V (GCC→GTC)	*astA* ←
	2,419,254	G→A	0.061	Q179* (CAG→TAG)	*yfcE* ←
	3,154,991	C→T	0.060	G76G (GGG→GGA)	*yqhC* ←
	3,337,061	T→C	0.060	K66E (AAA→GAA)	*mlaB* ←
	1,635,859	G→A	0.059	pseudogene (509/528 nt)	*nohQ* ←
	3,360,120	G→A	0.059	R308H (CGC→CAC)	*gltD* →
	4,244,741	C→T	0.058	R112H (CGC→CAC)	*malF* ←
	2,493,876	G→A	0.056	A176V (GCT→GTT)	*yfdX* ←
	2,693,690	C→T	0.056	T618T (ACG→ACA)	*purL* ←
	735,540	T→C	0.054	pseudogene (1408/1521 nt)	*rhsO* →
	1,975,163	T→C	0.054	A54A (GCA→GCG)	*cheA* ←
	2,201,321	C→T	0.054	T348I (ACA→ATA)	*yehI* →
	308,177	C→T	0.053	A386T (GCT→ACT)	*ecpC* ←
	1,466,495	C→T	0.053	pseudogene (1104/2513 nt)	*ydbA* →
	1,466,504	A→G	0.053	pseudogene (1113/2513 nt)	*ydbA* →
	1,089,760	T→C	0.052	Q33R (CAG→CGG)	*pgaB* ←
	2,110,222	A→G	0.052	V266V (GTT→GTC)	*rfbA* ←
	2,791,782	G→A	0.052	A170A (GCG→GCA)	*gabD* →
	1,003,408	T→G	0.051	Y174D (TAT→GAT)	*ycbU* →
	2,129,864	C→T	0.051	A339T (GCG→ACG)	*wcaD* ←
	2,967,828	G→A	0.051	L203L (CTG→TTG)	*ptsP* ←
	4,486,493	C→T	0.051	G92G (GGC→GGT)	*lptF* →
SAM2	3,571,062	A→G	0.050	F86F (TTT→TTC)	*glgX* ←
	12,661	C→G	1.000	R167G (CGT→GGT)	*dnaK* →
	594,727	C→A	1.000	R15L (CGC→CTC)	*cusS* ←
	3,101,306	G→T	1.000	I106I (ATC→ATA)	*yggN* ←
	3,133,461	G→A	1.000	A166V (GCA→GTA)	*yghS* ←
	3,536,061	Δ1 bp	1.000	coding (524/720 nt)	*ompR* ←
	3,816,605	Δ103 bp	1.000		*[yicC]*
	3,843,548	A→C	1.000	I81R (ATA→AGA)	*yicO* ←
	3,966,612	C→T	1.000	R66C (CGT→TGT)	*rho* →
	3,992,735	C→A	0.865	S528* (TCG→TAG)	*cyaA* →
	3,177,973	IS*1* (+) +9 bp	0.648	intergenic (-63/-134)	*nudF* ← / → *tolC*
	3,360,120	G→A	0.163	R308H (CGC→CAC)	*gltD* →
	4,296,060	C→T	0.159	intergenic (+266/+376)	*gltP* → / ← *yjcO*
	2,725,169	Δ1 bp	0.147	coding (578/1299 nt)	*kgtP* ←
	2,229,205	G→C	0.116	intergenic (+140/+233)	*yohP* → / ← *dusC*
	2,771,529	IS*3* (+) +3 bp	0.079	coding (382-384/468 nt)	*yfjT* →
	1,667,662	A→G	0.070	C301C (TGT→TGC)	*mlc* ←
	2,039,690	C→T	0.069	A71A (GCC→GCT)	*yedY* →
	2,888,245	T→C	0.066	E23G (GAA→GGA)	*cysH* ←
	529,421	T→C	0.059	intergenic (+291/-79)	*ybbD* → / → *ylbI*
	2,328,469	G→A	0.058	S443S (AGC→AGT)	*yfaQ* ←
	585,179	G→A	0.056	A152V (GCC→GTC)	*ompT* ←
	4,325,888	G→T	0.055	intergenic (-147/+511)	*yjdN* ← / ← *yjdM*
	2,335,324	A→G	0.052	S441P (TCG→CCG)	*yfaA* ←
	4,092,315	C→T	0.052	A18T (GCT→ACT)	*frvB* ←

SAM3	4,261,089	G→A	0.051	G164E (GGG→GAG)	*yjbM* →
	3,815,859	Δ82 bp	0.533		*[rph]* –*[rph]*
	3,815,824	G→T	0.277	intergenic (-56/+10)	*pyrE* ← / ← *rph*
	3,198,033	IS*186* (+) +6 bp :: Δ2 bp	0.258	coding (1604-1609/2841 nt)	*glnE* ←
	4,296,060	C→T	0.235	intergenic (+266/+376)	*gltP* → / ← *yjcO*
	3,815,809	Δ1 bp	0.138	intergenic (-41/+25)	*pyrE* ← / ← *rph*
	915,226	T→C	0.112	intergenic (-369/+126)	*ybjE* ← / ← *aqpZ*
	451,096	T→C	0.094	N172S (AAC→AGC)	*cyoA* ←
	1,708,718	G→A	0.071	A600A (GCG→GCA)	*rsxC* →
	1,869,932	IS*2* (+) +5 bp	0.064	coding (978-982/1284 nt)	*yeaH* →
	3,328,463	IS*4* (–) +11 bp	0.061	coding (243-253/477 nt)	*greA* ←
	594,727	C→A	0.055	R15L (CGC→CTC)	*cusS* ←
	1,821,249	T→C	0.055	intergenic (-35/+50)	*chbC* ← / ← *chbB*
SAM4	2,288,354	G→A	0.052	pseudogene (561/2525 nt)	*yejO* ←
	3,197,294	T→A	0.408	E783V (GAA→GTA)	*glnE* ←
	3,815,809	Δ1 bp	0.373	intergenic (-41/+25)	*pyrE* ← / ← *rph*
	3,815,859	Δ82 bp	0.367		*[rph]* –*[rph]*
	4,296,060	C→T	0.236	intergenic (+266/+376)	*gltP* → / ← *yjcO*
	3,992,588	(ATCAGCC)$_{2\rightarrow1}$	0.235	coding (1436-1442/2547 nt)	*cyaA* →
	1,907,503	IS*3* (–) +5 bp :: +T	0.178	coding (85-89/144 nt)	*yobF* ←
	4,181,669	A→G	0.164	E142G (GAG→GGG)	*rpoB* →
	3,485,966	IS*2* (+) +5 bp	0.129	intergenic (-148/-150)	*yhfA* ← / → *crp*
	3,897,059	C→A	0.129	L96I (CTC→ATC)	*yieH* →
	224,808	T→C	0.089	noncoding (1038/1542 nt)	*rrsH* →
	900,123	G→A	0.080	V151V (GTC→GTT)	*artJ* ←
	293,326	A→G	0.071	I198T (ATC→ACC)	*yagL* ←
	1,466,210	G→A	0.068	pseudogene (819/2513 nt)	*ydbA* →
	1,287,037	C→T	0.061	intergenic (+511/+29)	*narI* → / ← *rttR*
	1,269,450	C→T	0.058	intergenic (+61/+253)	*rdlA* → / ← *ldrB*
	1,269,451	G→T	0.058	intergenic (+62/+252)	*rdlA* → / ← *ldrB*
	1,496,764	C→T	0.054	intergenic (+133/-92)	*ydcJ* → / → *opgD*
SAM5	1,466,201	T→C	0.051	pseudogene (810/2513 nt)	*ydbA* →
	594,727	C→A	1.000	R15L (CGC→CTC)	*cusS* ←
	710,620	C→A	1.000	K9N (AAG→AAT)	*fur* ←
	3,440,186	C→A	1.000	V282L (GTA→TTA)	*rpoA* ←
	3,536,041	G→A	1.000	R182C (CGC→TGC)	*ompR* ←
	3,966,604	G→T	1.000	G63V (GGT→GTT)	*rho* →
	4,232,641	C→A	0.318	R198L (CGT→CTT)	*lysC* ←
	1,213,820	G→C	0.279	D80E (GAC→GAG)	*bluR* ←
	3,359,461	Δ1 bp	0.226	coding (264/1419 nt)	*gltD* →
	3,815,809	Δ1 bp	0.196	intergenic (-41/+25)	*pyrE* ← / ← *rph*
	4,296,060	C→T	0.193	intergenic (+266/+376)	*gltP* → / ← *yjcO*
	1,153,243	G→T	0.154	intergenic (+63/-57)	*fabF* → / → *pabC*
	3,992,588	(ATCAGCC)$_{2\rightarrow1}$	0.141	coding (1436-1442/2547 nt)	*cyaA* →
	1,734,295	G→A	0.087	intergenic (-194/-140)	*grxD* ← / → *mepH*
	1,783,972	A→G	0.080	T314T (ACA→ACG)	*fadK* →
	3,638,173	C→T	0.077	R178C (CGT→TGT)	*pitA* →
	1,529,496	T→C	0.074	pseudogene (1595/2037 nt)	*rhsE* →
	1,417,872	C→T	0.071	C46Y (TGT→TAT)	*ydaE* ←
	1,118,073	G→C	0.069	T135R (ACG→AGG)	*yceI* ←
	1,406,828	C→T	0.066	R323H (CGC→CAC)	*ydaM* ←
	1,415,602	T→A	0.061	I595I (ATA→ATT)	*recE* ←
	4,214,313	G→T	0.061	V12F (GTC→TTC)	*metA* →
	913,671	C→T	0.060	W48* (TGG→TGA)	*hcp* ←
	2,480,006	G→A	0.060	D269N (GAT→AAT)	*dsdA* →
	1,900,663	C→A	0.059	G308V (GGT→GTT)	*yoaE* ←
	777,885	C→A	0.058	A49E (GCG→GAG)	*tolB* →
	1,136,015	C→T	0.058	T151M (ACG→ATG)	*flgH* →
	1,495,623	G→A	0.058	R112R (CGG→CGA)	*ydcJ* →
	2,545,513	G→A	0.058	Q33* (CAA→TAA)	*murR* ←
	2,560,599	T→C	0.058	L115L (TTG→CTG)	*yffL* →
	2,278,939	G→T	0.057	R274R (CGC→CGA)	*bcr* ←
	3,614,258	G→A	0.057	D198N (GAT→AAT)	*nikA* →
	372,696	C→T	0.056	S194S (AGC→AGT)	*mhpD* →
	1,244,206	A→G	0.056	L108L (TTA→CTA)	*ycgR* ←
	3,915,514	C→T	0.056	intergenic (-314/+39)	*glmU* ← / ← *atpC*
	4,522,674	T→C	0.056	K474R (AAG→AGG)	*yjhG* ←
SAM6	2,179,710	A→G	0.055	Y297C (TAT→TGT)	*yegT* →
	594,727	C→A	1.000	R15L (CGC→CTC)	*cusS* ←
	710,620	C→A	1.000	K9N (AAG→AAT)	*fur* ←
	1,428,765	T→C	1.000	intergenic (-39/-30)	*insH1* ← / → *lomR*
	3,440,186	C→A	1.000	V282L (GTA→TTA)	*rpoA* ←
	3,536,041	G→A	1.000	R182C (CGC→TGC)	*ompR* ←
	3,815,801	Δ1 bp	1.000	intergenic (-33/+33)	*pyrE* ← / ← *rph*
	3,966,604	G→T	1.000	G63V (GGT→GTT)	*rho* →
	1,212,080:1	+C	1.000	intergenic (-77/+623)	*iraM* ← / ← *ycgX*
	1,213,820	G→C	0.627	D80E (GAC→GAG)	*bluR* ←

	3,359,461	Δ1 bp	0.590	coding (264/1419 nt)	*gltD* →
	4,296,060	C→T	0.248	intergenic (+266/+376)	*gltP* → / ← *yjcO*
	580,011	IS*3* (–) +4 bp :: +TC	0.156	coding (132-135/207 nt)	*ybcW* →
	4,233,625	IS*5* (+) +4 bp	0.124	intergenic (-392/-130)	*lysC* ← / → *pgi*
	3,815,809	Δ1 bp	0.117	intergenic (-41/+25)	*pyrE* ← / ← *rph*
	4,153,616	C→T	0.067	intergenic (-518/+80)	*ppc* ← / ← *argE*
	2,630,544	G→T	0.057	A73A (GCG→GCT)	*yfgI* →
	1,463,083	G→A	0.056	R320H (CGC→CAC)	*paaK* →
SAM7	847,955	G→A	0.000	A17V (GCG→GTG)	*glnH* ←
