# Supplementary material for: It Takes Two to Make a Thing Go Right: Epistasis, Two-Component Response Systems, and Bacterial Adaptation
Source: Microorganisms. 2024 Sep 30;12(10):2000. doi: 10.3390/microorganisms12102000 (PMC11510482; doi:10.3390/microorganisms12102000)
Supplement: Supplementary file 1 [file microorganisms-12-02000-s001.zip › TableS2.pdf]

Table S2 - Two-way ANOVA with pairwise comparisons of 24-hour growth data. MIC values have been included for each population and data has been remove for all populations once they have reached their MIC.

[Silver nitrate]		0 ng/mL		5 ng/mL		50 ng/mL		60 ng/mL		70 ng/mL		80 ng/mL		90 ng/mL		100 ng/mL		250 ng/mL		500 ng/mL		750 ng/mL	
MIC								MIC WT		MIC R15L				MIC SAM2		MIC SAM5		MIC SAM3		MIC SAM4		MIC SAM1 and SAM6	
Statistical comparaison		vs WT		vs WT		vs WT		vs SAM1		vs SAM1		vs SAM1		vs SAM1		vs SAM1		vs SAM1		vs SAM1			
Hours	Population	Summary	Adjusted P V	Summary	Adjusted P V	Summary	Adjusted P V	Summary	Adjusted P V	Summary	Adjusted P V	Summary	Adjusted P V	Summary	Adjusted P V	Summary	Adjusted P V	Summary	Adjusted P V	Summary	Adjusted P V	Summary	Adjusted P Value
t=0	WT																						
	R15L	*	0.019	ns	0.2258	ns	0.0952	*	0.0181														
	SAM1	*	0.0344	ns	0.1922	ns	0.1661																
	SAM2	*	0.0131	ns	0.2607	ns	0.3518	ns	0.9731	ns	>0.9999	ns	0.7815	ns	0.6366	ns	0.6724	ns	0.724	ns	0.6287		
	SAM3	*	0.0293	ns	0.0983	ns	0.0875	*	0.0117	***	0.0006	*	0.0108	ns	0.0678	*	0.0138						
	SAM4	ns	0.0724	ns	0.1419	ns	0.056	ns	0.9997	ns	0.6872	ns	0.9997	ns	0.998	ns	0.411	ns	0.9716				
	SAM5	ns	0.2565	ns	0.4027	ns	0.5469	**	0.0026	ns	0.0639	*	0.0434	**	0.0086								
	SAM6	*	0.0358	ns	0.2442	ns	0.2231	ns	0.997	ns	0.9437	ns	0.9985	ns	0.9889	ns	0.9913	ns	>0.9999	ns	>0.9999		
t=1	WT																						
	R15L	*	0.0197	**	0.0059	ns	0.4747	*	0.02														
	SAM1	ns	0.2226	ns	0.4619	***	0.0007																
	SAM2	ns	0.0969	ns	0.9856	ns	0.8839	ns	0.4854	ns	0.8932	ns	0.3674										
	SAM3	ns	0.7113	ns	0.2354	*	0.0225	*	0.041	ns	0.1964	ns	0.3717	ns	0.4557	ns	>0.9999						
	SAM4	ns	0.9056	ns	0.6154	ns	0.0846	ns	0.1071	*	0.018	*	0.0162	ns	0.589	ns	0.1328	ns	0.1274				
	SAM5	ns	0.1924	ns	0.4102	**	0.0055	ns	0.4991	ns	0.0595	ns	0.6448	ns	>0.9999								
	SAM6	ns	0.0796	ns	>0.9999	ns	0.0607	ns	0.1133	ns	0.0853	ns	0.2443	ns	0.2739	ns	0.9568	ns	0.9957	ns	0.9995		
t=2	WT																						
	R15L	**	0.0057	**	0.0029	ns	0.5344	*	0.0272														
	SAM1	*	0.048	ns	0.1573	***	0.0004																
	SAM2	*	0.0136	ns	0.9111	ns	0.668	*	0.048	ns	0.105	**	0.0023										
	SAM3	ns	0.1165	ns	0.0568	**	0.0069	*	0.0332	*	0.0125	ns	0.0769	*	0.0166	ns	0.3816						
	SAM4	*	0.0326	ns	0.9427	ns	0.0592	ns	>0.9999	ns	>0.9999	ns	0.7659	ns	0.2117	ns	0.9933	ns	0.2535				
	SAM5	*	0.0156	ns	0.27	**	0.0063	ns	0.4479	ns	0.9293	ns	0.0613	*	0.0257								
	SAM6	*	0.023	ns	0.71	*	0.0336	ns	0.0881	**	0.0038	*	0.0253	**	0.0079	ns	0.2911	ns	0.9909	ns	0.9995		
t=3	WT																						
	R15L	***	0.001	***	0.0004	ns	0.5228	*	0.0263														
	SAM1	**	0.0084	*	0.0254	***	0.0002																
	SAM2	**	0.0068	ns	0.9707	ns	0.5581	**	0.0065	**	0.0013	****	<0.0001										
	SAM3	*	0.0189	**	0.0081	***	0.0005	*	0.0361	**	0.0037	**	0.0092	*	0.0154	ns	0.2453						
	SAM4	**	0.0042	ns	0.9109	ns	0.0599	ns	0.0832	*	0.0342	***	0.0009	*	0.0471	ns	0.3509	ns	0.8922				
	SAM5	**	0.0033	ns	0.0748	**	0.0088	*	0.0497	*	0.0192	***	0.0005	*	0.0208								
	SAM6	*	0.0118	ns	0.0597	*	0.0145	ns	0.0555	**	0.0054	***	0.0009	**	0.009	ns	0.1429	ns	0.3688	ns	0.6128		
t=4	WT																						
	R15L	**	0.0024	****	<0.0001	ns	0.5092	*	0.0258														
	SAM1	*	0.019	**	0.002	**	0.0034																
	SAM2	**	0.004	ns	>0.9999	ns	0.4355	*	0.0122	**	0.0031	****	<0.0001										
	SAM3	***	0.0008	**	0.0033	***	0.0001	*	0.0388	*	0.0159	***	0.0008	ns	0.1088	ns	0.1697						
	SAM4	**	0.0025	ns	0.164	*	0.0435	*	0.0374	*	0.022	***	0.0004	ns	0.0989	ns	0.1588	*	0.0363				
	SAM5	***	0.0008	**	0.0031	**	0.0092	*	0.0369	*	0.0253	****	<0.0001	ns	0.0847								
	SAM6	*	0.0468	*	0.02	**	0.0033	ns	0.0706	*	0.0168	****	<0.0001	ns	0.0763	ns	0.1164	*	0.0255	ns	0.0929		
t=5	WT																						
	R15L	*	0.0108	***	0.0003	ns	0.6203	*	0.0134														
	SAM1	*	0.0177	**	0.0056	**	0.0039																
	SAM2	*	0.0137	ns	0.6166	ns	0.3686	**	0.0077	**	0.0053	***	0.0003										
	SAM3	***	0.0003	****	<0.0001	****	<0.0001	*	0.0211	*	0.02	***	0.0002	ns	0.138	ns	0.1396						
	SAM4	**	0.0032	ns	0.0888	ns	0.0522	*	0.0147	*	0.0177	**	0.0021	ns	0.0966	ns	0.1036	**	0.0084				
	SAM5	*	0.0218	**	0.0013	**	0.0066	*	0.0166	*	0.0196	**	0.0015	ns	0.0909								
	SAM6	ns	0.8298	**	0.005	***	0.001	ns	0.1174	*	0.0138	***	0.0001	ns	0.0917	ns	0.0951	**	0.0031	*	0.0123		
t=6	WT																						
	R15L	*	0.0413	****	<0.0001	ns	0.9633	**	0.0085														
	SAM1	ns	0.211	**	0.0012	**	0.0034																
	SAM2	ns	0.1531	ns	0.3038	ns	0.3221	*	0.0107	***	0.0007	***	0.0004										
	SAM3	**	0.0058	***	0.0002	***	0.0002	*	0.0219	*	0.01	****	<0.0001	ns	0.1156	ns	0.1133						
	SAM4	ns	0.0502	ns	0.2702	*	0.0175	*	0.0136	**	0.0096	**	0.0015	ns	0.0746	ns	0.069	**	0.0063				
	SAM5	ns	0.5292	***	0.0001	**	0.0057	*	0.0147	**	0.0087	***	0.001	ns	0.0724								
	SAM6	ns	0.3009	***	0.0001	***	0.0002	ns	0.3169	**	0.0032	***	0.0007	ns	0.0779	ns	0.0757	**	0.0035	**	0.0012		
t=7	WT																						
	R15L	*	0.0265	****	<0.0001	ns	0.9997	**	0.0068														
	SAM1	ns	0.2308	**	0.0074	**	0.0022																
	SAM2	ns	0.2151	ns	0.352	ns	0.2858	*	0.0142	****	<0.0001	**	0.0012										
	SAM3	**	0.0062	***	0.0003	***	0.0003	*	0.0284	**	0.0016	***	0.0003	ns	0.0618	ns	0.0694						
	SAM4	ns	0.0778	*	0.0165	*	0.0405	*	0.0151	**	0.0035	**	0.002	*	0.0343	*	0.0234	**	0.0063				
	SAM5	ns	0.9465	***	0.0008	**	0.007	*	0.0152	***	0.0005	**	0.0016	*	0.0336								
	SAM6	ns	0.284	**	0.0023	*	0.0105	ns	0.5654	***	0.0008	**	0.0084	*	0.0393	*	0.0397	**	0.0065	**	0.0019		
t=8	WT																						
	R15L	*	0.0313	***	0.0006	ns	0.9729	**	0.0064														
	SAM1	ns	0.1199	ns	0.0833	**	0.0017																
	SAM2	ns	0.3622	*	0.0493	ns	0.2834	*	0.0167	***	0.0001	**	0.003										
	SAM3	**	0.0061	***	0.0008	****	<0.0001	*	0.0473	***	0.0001	**	0.0019	*	0.0258	*	0.042						
	SAM4	ns	0.2459	**	0.0091	ns	0.4283	*	0.0186	***	0.0009	**	0.0037	*	0.0117	**	0.0055	**	0.0021				
	SAM5	ns	0.976	***	0.0005	**	0.0077	*	0.0153	***	0.0007	**	0.0034	*	0.0116								
	SAM6	ns	0.9989	**	0.0078	*	0.0192	ns	0.9966	*	0.0138	*	0.0282	*	0.015	*	0.0109	**	0.0016	**	0.0013		
t=9	WT																						
	R15L	ns	>0.9999	**	0.0014	ns	0.925	**	0.0057														
	SAM1	ns	0.9854	ns	>0.9999	***	0.0003																
	SAM2	ns	>0.9999	ns	0.1684	ns	0.2931	**	0.008	**	0.0034	**	0.0036										
	SAM3	**	0.0083	**	0.0022	***	0.0002	ns	0.0623	**	0.0036	**	0.0059	***	0.0001	*	0.0298						
	SAM4	ns	0.9691	ns	0.448	ns	>0.9999	*	0.0154	****	<0.0001	**	0.0043	***	0.0006	*	0.0352	**	0.0016				
	SAM5	ns	>0.9999	ns	>0.9999	**	0.0026	**	0.0041	*	0.0163	**	0.0041	***	0.0007								

	SAM6	ns	0.9986	ns	0.784	*	0.0166	ns	0.9805	*	0.0392	ns	0.0588	****	<0.0001	**	0.0035	***	0.0002	**	0.0024
t=10	WT																				
	R15L	ns	>0.9999	*	0.0234	ns	0.8927	ns	0.0893												
	SAM1	ns	0.997	ns	0.9804	****	<0.0001														
	SAM2	ns	0.5702	ns	0.0517	ns	0.3212	***	0.0006	ns	0.0606	**	0.0018								
	SAM3	ns	0.0536	ns	0.9558	***	0.0004	ns	0.1486	ns	0.0505	ns	0.0504	**	0.0065	**	0.0091				
	SAM4	ns	0.8759	ns	0.5863	ns	0.9068	*	0.0124	****	<0.0001	**	0.0031	**	0.0017	ns	0.1912	****	<0.0001		
	SAM5	ns	0.9872	ns	0.7076	****	<0.0001	*	0.0279	ns	0.0634	**	0.0028	**	0.0018						
	SAM6	ns	0.9999	ns	0.9935	**	0.0042	ns	0.8306	ns	0.1228	ns	0.1668	**	0.001	*	0.0479	***	0.0004	**	0.0074
t=11	WT																				
	R15L	ns	>0.9999	ns	0.1857	ns	0.8776	ns	0.1885												
	SAM1	ns	0.992	ns	0.9108	**	0.0038														
	SAM2	ns	0.294	**	0.0046	ns	0.4103	**	0.0012	ns	0.294	***	0.0008								
	SAM3	ns	0.9987	ns	0.0924	**	0.0052	ns	0.8122	ns	0.4329	ns	0.9689	ns	0.093	ns	0.9915				
	SAM4	ns	0.696	ns	0.1286	ns	0.7806	*	0.0132	****	<0.0001	**	0.0035	***	0.0008	ns	0.5961	****	<0.0001		
	SAM5	ns	0.884	ns	0.1011	**	0.0044	ns	0.3014	ns	0.4471	**	0.0033	**	0.0011						
	SAM6	ns	0.985	ns	0.9685	**	0.0044	ns	0.8544	ns	0.2395	ns	0.0661	**	0.0058	ns	0.3417	**	0.0021	ns	0.1139
t=12	WT																				
	R15L	ns	0.9947	ns	0.3203	ns	0.8464	ns	0.6019												
	SAM1	ns	0.9993	ns	0.9854	*	0.0323														
	SAM2	ns	0.2034	***	0.0007	ns	0.6064	*	0.0245	ns	0.3936	****	<0.0001								
	SAM3	ns	>0.9999	ns	0.2107	*	0.0292	ns	0.9944	ns	0.9635	ns	>0.9999	ns	0.8315	ns	0.2865				
	SAM4	ns	0.6356	ns	0.0755	ns	0.6721	*	0.0149	****	<0.0001	**	0.0035	***	0.0003	ns	0.8241	**	0.0022		
	SAM5	ns	0.8217	ns	0.0724	*	0.0389	ns	0.87	ns	>0.9999	**	0.0033	***	0.001						
	SAM6	ns	0.9379	ns	0.9698	*	0.0215	ns	0.9163	ns	0.5188	ns	0.2856	ns	>0.9999	ns	0.9978	**	0.0025	ns	0.8903
t=13	WT																				
	R15L	ns	0.5054	ns	0.9819	ns	0.7984	ns	0.7943												
	SAM1	ns	0.9941	ns	0.9991	ns	0.1112														
	SAM2	ns	0.1266	***	0.0003	ns	0.8853	ns	0.137	ns	0.4332	****	<0.0001								
	SAM3	ns	>0.9999	ns	0.3435	ns	0.1063	ns	0.9485	ns	0.8485	ns	>0.9999	ns	0.7396	ns	0.2539				
	SAM4	ns	0.4991	*	0.0396	ns	0.6153	*	0.0126	****	<0.0001	**	0.0035	****	<0.0001	ns	0.9542	**	0.0012		
	SAM5	ns	0.6965	ns	0.0888	ns	0.1281	ns	0.9325	ns	0.5061	**	0.0034	***	0.0006						
	SAM6	ns	0.8419	ns	0.9887	ns	0.1186	ns	0.8913	ns	0.3627	ns	0.3229	ns	0.7648	ns	0.9588	ns	0.062	ns	0.9129
t=14	WT																				
	R15L	ns	0.2185	ns	0.4607	ns	0.7258	ns	0.8644												
	SAM1	ns	0.9986	ns	>0.9999	ns	0.3119														
	SAM2	ns	0.0872	***	0.0006	ns	0.9985	ns	0.1904	ns	0.5292	***	0.0007								
	SAM3	ns	>0.9999	ns	0.4815	ns	0.3018	ns	0.9514	ns	0.9295	ns	>0.9999	ns	0.765	ns	0.2407				
	SAM4	ns	0.4382	*	0.0359	ns	0.5925	**	0.0079	****	<0.0001	**	0.003	***	0.0004	ns	0.9785	***	0.0002		
	SAM5	ns	0.5974	ns	0.1466	ns	0.3519	ns	0.9411	ns	0.2788	**	0.0032	***	0.0004						
	SAM6	ns	0.7264	ns	>0.9999	ns	0.3652	ns	0.9114	ns	0.4119	ns	0.3419	ns	0.8659	ns	0.9723	ns	0.6474	ns	0.8792
t=15	WT																				
	R15L	ns	0.1882	ns	0.2949	ns	0.7023	ns	0.9342												
	SAM1	ns	0.9994	ns	>0.9999	ns	0.5919														
	SAM2	ns	0.0556	***	0.0008	ns	>0.9999	ns	0.2912	ns	0.5627	*	0.0117								
	SAM3	ns	>0.9999	ns	0.5193	ns	0.5838	ns	0.9471	ns	0.8301	ns	>0.9999	ns	0.7638	ns	0.2815				
	SAM4	ns	0.3671	ns	0.0542	ns	0.5968	**	0.0045	****	<0.0001	**	0.0012	**	0.0065	ns	0.9996	***	0.0001		
	SAM5	ns	0.5052	ns	0.2076	ns	0.6468	ns	0.9582	ns	0.2476	**	0.0034	***	0.0005						
	SAM6	ns	0.5917	ns	>0.9999	ns	0.6885	ns	0.9385	ns	0.3362	ns	0.3727	ns	0.8851	ns	0.9841	ns	0.9995	ns	0.9409
t=16	WT																				
	R15L	ns	0.2068	ns	0.1881	ns	0.6446	ns	0.8631												
	SAM1	ns	>0.9999	ns	>0.9999	ns	0.7388														
	SAM2	ns	0.1191	***	0.0009	ns	>0.9999	ns	0.4856	ns	0.6223	*	0.0285								
	SAM3	ns	0.9996	ns	0.6222	ns	0.7373	ns	0.9347	ns	0.7924	ns	>0.9999	ns	0.6999	ns	0.308				
	SAM4	ns	0.6476	ns	0.2723	ns	0.6487	*	0.0212	****	<0.0001	***	0.0004	*	0.0364	ns	0.9911	**	0.0016		
	SAM5	ns	0.7689	ns	0.3681	ns	0.7945	ns	0.9616	ns	0.1923	**	0.0046	***	0.0004						
	SAM6	ns	0.6117	ns	>0.9999	ns	0.839	ns	0.969	ns	0.3202	ns	0.4577	ns	0.9306	ns	0.9974	ns	0.9941	ns	0.9702
t=17	WT																				
	R15L	ns	0.3094	ns	0.1198	ns	0.7278	ns	0.6867												
	SAM1	ns	0.9727	ns	0.9993	ns	0.8145														
	SAM2	ns	0.2742	**	0.0021	ns	0.9999	ns	0.601	ns	0.6579	*	0.0287								
	SAM3	ns	0.989	ns	0.713	ns	0.8136	ns	0.9375	ns	0.7005	ns	>0.9999	ns	0.7147	ns	0.3066				
	SAM4	ns	0.9488	ns	0.6271	ns	0.8072	ns	0.1536	***	0.0003	**	0.0017	ns	0.1544	ns	0.0595	*	0.0325		
	SAM5	ns	0.9835	ns	0.5305	ns	0.8586	ns	0.9725	ns	0.1684	**	0.0049	****	<0.0001						
	SAM6	ns	0.6708	ns	>0.9999	ns	0.9078	ns	0.9886	ns	0.2493	ns	0.4589	ns	0.9853	ns	0.9994	ns	0.9857	ns	0.986
t=18	WT																				
	R15L	ns	0.5489	ns	0.0992	ns	0.7324	ns	0.6427												
	SAM1	ns	0.8493	ns	0.996	ns	0.8892														
	SAM2	ns	0.5984	**	0.0033	ns	0.9992	ns	0.649	ns	0.6932	*	0.0365								
	SAM3	ns	0.9226	ns	0.7484	ns	0.8938	ns	0.9142	ns	0.7227	ns	>0.9999	ns	0.7623	ns	0.3354				
	SAM4	ns	>0.9999	ns	0.8155	ns	0.9635	ns	0.488	**	0.008	*	0.0256	ns	0.5056	ns	0.0896	ns	0.7762		
	SAM5	ns	>0.9999	ns	0.6975	ns	0.9207	ns	0.9796	ns	0.1174	**	0.0051	**	0.0021						
	SAM6	ns	0.8378	ns	0.9998	ns	0.9617	ns	0.9988	ns	0.2833	ns	0.5166	ns	0.9964	ns	0.9998	ns	0.9728	ns	0.9933
t=19	WT																				
	R15L	ns	0.7583	ns	0.0887	ns	0.7216	ns	0.6283												
	SAM1	ns	0.7584	ns	0.9951	ns	0.9655														
	SAM2	ns	0.8566	**	0.0046	ns	0.9921	ns	0.7129	ns	0.7103	*	0.0443								
	SAM3	ns	0.8421	ns	0.7885	ns	0.9656	ns	0.933	ns	0.7372	ns	>0.9999	ns	0.8062	ns	0.3374				
	SAM4	ns	0.9997	ns	0.9543	ns	0.9983	ns	0.8354	ns	0.0537	ns	0.1387	ns	0.9785	ns	0.1377	ns	0.4174		
	SAM5	ns	0.9962	ns	0.766	ns	0.9771	ns	0.9862	ns	0.1113	**	0.0028	*	0.0468						
	SAM6	ns	0.9477	ns	0.9858	ns	0.9961	ns	>0.9999	ns	0.3699	ns	0.6107	ns	>0.9999	ns	>0.9999	ns	0.9656	ns	>0.9999
t=20	WT																				
	R15L	ns	0.8626	ns	0.0799	ns	0.7288	ns	0.6273												
	SAM1	ns	0.6863	ns	0.9968	ns	0.9944														

	SAM2	ns	0.9692	**	0.0056	ns	0.9644	ns	0.7431	ns	0.7208	*	0.0483								
	SAM3	ns	0.7711	ns	0.7928	ns	0.995	ns	0.9153	ns	0.6773	ns	>0.9999	ns	0.8006	ns	0.3447				
	SAM4	ns	0.9939	ns	0.988	ns	>0.9999	ns	0.9931	ns	0.2076	ns	0.5243	ns	0.97	ns	0.1493	ns	0.1058		
	SAM5	ns	0.9782	ns	0.7925	ns	0.9962	ns	0.9893	ns	0.0925	***	0.0008	ns	0.1979						
	SAM6	ns	0.9804	ns	0.7751	ns	>0.9999	ns	>0.9999	ns	0.4994	ns	0.6581	ns	>0.9999	ns	>0.9999	ns	0.9501	ns	>0.9999
t=21	WT																				
	R15L	ns	0.9284	ns	0.0566	ns	0.7161	ns	0.6688												
	SAM1	ns	0.6215	ns	0.9994	ns	>0.9999														
	SAM2	ns	0.9982	**	0.0066	ns	0.8795	ns	0.7705	ns	0.7274	ns	0.0509								
	SAM3	ns	0.7163	ns	0.8742	ns	>0.9999	ns	0.9045	ns	0.7226	ns	>0.9999	ns	0.8756	ns	0.3441				
	SAM4	ns	0.9823	ns	0.986	ns	0.9998	ns	>0.9999	ns	0.6175	ns	>0.9999	ns	0.3792	ns	0.1618	ns	0.0887		
	SAM5	ns	0.9451	ns	0.7354	ns	>0.9999	ns	0.9906	ns	0.1098	ns	0.0565	ns	0.4471						
	SAM6	ns	0.9925	ns	0.4592	ns	>0.9999	ns	0.9976	ns	0.6104	ns	0.6517	ns	>0.9999	ns	>0.9999	ns	0.9302	ns	0.9999
t=22	WT																				
	R15L	ns	0.9699	*	0.038	ns	0.7389	ns	0.6672												
	SAM1	ns	0.561	ns	0.998	ns	0.9945														
	SAM2	ns	>0.9999	*	0.0107	ns	0.6233	ns	0.7822	ns	0.7173	ns	0.0559								
	SAM3	ns	0.6567	ns	0.8829	ns	0.9975	ns	0.9133	ns	0.7177	ns	0.9999	ns	0.8688	ns	0.3555				
	SAM4	ns	0.9664	ns	0.9939	ns	0.9857	ns	0.9646	ns	0.9647	ns	0.899	ns	0.1391	ns	0.1707	ns	0.0824		
	SAM5	ns	0.909	ns	0.7406	ns	0.9979	ns	0.989	ns	0.142	ns	0.245	ns	0.5522						
	SAM6	ns	0.9989	ns	0.367	ns	0.9023	ns	0.9787	ns	0.6728	ns	0.6529	ns	0.9994	ns	>0.9999	ns	0.9074	ns	0.9988
t=23	WT																				
	R15L	ns	0.9877	ns	0.0567	ns	0.8719	ns	0.7685												
	SAM1	ns	0.522	ns	0.9981	ns	0.2675														
	SAM2	ns	>0.9999	*	0.0161	ns	0.3654	ns	0.7768	ns	0.721	ns	0.0679								
	SAM3	ns	0.6234	ns	0.9429	ns	0.3233	ns	0.9058	ns	0.7249	ns	0.9999	ns	0.8834	ns	0.3529				
	SAM4	ns	0.9474	ns	0.9981	ns	0.9865	ns	0.8536	ns	>0.9999	ns	0.8507	ns	0.1368	ns	0.184	ns	0.1267		
	SAM5	ns	0.8767	ns	0.8384	ns	0.3	ns	0.987	ns	0.2004	ns	0.4981	ns	0.5292						
	SAM6	ns	>0.9999	ns	0.4699	ns	0.1437	ns	0.9694	ns	0.6842	ns	0.6155	ns	0.9908	ns	>0.9999	ns	0.8819	ns	0.993
t=24	WT																				
	R15L	ns	0.9992	ns	0.1529	ns	0.9	ns	0.7998												
	SAM1	ns	0.4614	ns	0.9967	ns	0.1635														
	SAM2	ns	0.9998	*	0.0333	ns	0.3362	ns	0.7824	ns	0.7093	ns	0.1035								
	SAM3	ns	0.5465	ns	0.9749	ns	0.2033	ns	0.9098	ns	0.7316	ns	0.9995	ns	0.8829	ns	0.3581				
	SAM4	ns	0.8962	ns	0.9998	ns	0.9895	ns	0.85	ns	>0.9999	ns	0.8591	ns	0.1449	ns	0.1834	ns	0.1108		
	SAM5	ns	0.8078	ns	0.9349	ns	0.1815	ns	0.9831	ns	0.2856	ns	0.7437	ns	0.5233	****					
	SAM6	ns	>0.9999	ns	0.5187	ns	0.0835	ns	0.9577	ns	0.7123	ns	0.5808	ns	0.9714	ns	0.9995	ns	0.8547	ns	0.9808
