# Supplementary material for: It Takes Two to Make a Thing Go Right: Epistasis, Two-Component Response Systems, and Bacterial Adaptation
Source: Microorganisms. 2024 Sep 30;12(10):2000. doi: 10.3390/microorganisms12102000 (PMC11510482; doi:10.3390/microorganisms12102000)
Supplement: Supplementary file 1 [file microorganisms-12-02000-s001.zip › TableS3.pdf]

Table S3 - One-way ANOVA with multiple pairwise comparisons of relative fitness data

[Silver Nitrate]	0 ng/mL		5 ng/mL		50 ng/mL		60 ng/mL		70 ng/mL		80 ng/mL		90 ng/mL		100 ng/mL		250 ng/mL		500 ng/mL	
	Summary	Adjusted P Value	Summary	Adjusted P Value	Summary	Adjusted P Value	Summary	Adjusted P Value	Summary	Adjusted P Value	Summary	Adjusted P Value	Summary	Adjusted P Value	Summary	Adjusted P Value	Summary	Adjusted P Value	Summary	Adjusted P Value
WT vs. R15L	ns	0.4927	ns	0.3959	**	0.0044	****	<0.0001	ns	>0.9999	ns	>0.9999	ns	>0.9999	ns	>0.9999	ns	>0.9999	ns	>0.9999
WT vs. SAM1	ns	0.3892	ns	0.9047	**	0.0035	****	<0.0001	****	<0.0001	****	<0.0001	****	<0.0001	****	<0.0001	****	<0.0001	****	<0.0001
WT vs. SAM2	ns	0.8795	ns	0.6335	ns	0.1807	*	0.0286	ns	0.9042	ns	0.722	ns	>0.9999	ns	>0.9999	ns	>0.9999	ns	>0.9999
WT vs. SAM3	***	0.0003	ns	0.98	ns	0.5296	****	<0.0001	****	<0.0001	****	<0.0001	****	<0.0001	****	<0.0001	ns	>0.9999	ns	>0.9999
WT vs. SAM4	ns	0.7399	ns	0.9831	ns	0.9387	**	0.0048	ns	0.0811	****	<0.0001	***	0.0002	****	<0.0001	****	<0.0001	ns	>0.9999
WT vs. SAM5	ns	0.7074	ns	0.6877	**	0.0016	****	<0.0001	****	<0.0001	ns	0.6389	ns	0.9863	ns	>0.9999	ns	0.8978	ns	>0.9999
WT vs. SAM6	ns	>0.9999	ns	0.509	ns	0.1608	****	<0.0001	****	<0.0001	****	<0.0001	****	<0.0001	****	<0.0001	****	<0.0001	****	<0.0001
R15L vs. SAM1	ns	>0.9999	*	0.0174	ns	>0.9999	**	0.0068	****	<0.0001	****	<0.0001	****	<0.0001	****	<0.0001	****	<0.0001	****	<0.0001
R15L vs. SAM2	ns	0.9982	**	0.0031	ns	0.9085	*	0.038	ns	0.9042	ns	0.722	ns	>0.9999	ns	>0.9999	ns	>0.9999	ns	>0.9999
R15L vs. SAM3	ns	0.1839	ns	0.9363	ns	0.56	ns	>0.9999	****	<0.0001	****	<0.0001	****	<0.0001	****	<0.0001	ns	>0.9999	ns	>0.9999
R15L vs. SAM4	ns	>0.9999	ns	0.0505	ns	0.145	ns	0.1546	ns	0.0811	****	<0.0001	***	0.0002	****	<0.0001	****	<0.0001	ns	>0.9999
R15L vs. SAM5	ns	>0.9999	**	0.0042	ns	>0.9999	ns	0.8259	****	<0.0001	ns	0.6389	ns	0.9863	ns	>0.9999	ns	0.8978	ns	>0.9999
R15L vs. SAM6	ns	0.7508	**	0.0016	ns	0.9257	ns	0.3947	****	<0.0001	****	<0.0001	****	<0.0001	****	<0.0001	****	<0.0001	****	<0.0001
SAM1 vs. SAM2	ns	0.9929	ns	0.9997	ns	0.884	****	<0.0001	****	<0.0001	****	<0.0001	****	<0.0001	****	<0.0001	****	<0.0001	****	<0.0001
SAM1 vs. SAM3	ns	0.2549	ns	0.3354	ns	0.5157	*	0.0179	**	0.0076	**	0.0025	**	0.0019	ns	0.2893	****	<0.0001	****	<0.0001
SAM1 vs. SAM4	ns	0.9995	ns	>0.9999	ns	0.1245	****	<0.0001	****	<0.0001	****	<0.0001	****	<0.0001	*	0.0396	****	<0.0001	****	<0.0001
SAM1 vs. SAM5	ns	0.9997	ns	0.9999	ns	>0.9999	****	<0.0001	ns	0.29	****	<0.0001	****	<0.0001	****	<0.0001	****	<0.0001	****	<0.0001
SAM1 vs. SAM6	ns	0.6497	ns	0.9974	ns	0.9041	ns	0.7719	**	0.0026	****	<0.0001	***	0.0002	**	0.0034	****	<0.0001	****	<0.0001
SAM2 vs. SAM3	*	0.0355	ns	0.1185	ns	0.9986	*	0.0154	****	<0.0001	****	<0.0001	****	<0.0001	****	<0.0001	ns	>0.9999	ns	>0.9999
SAM2 vs. SAM4	ns	>0.9999	ns	0.9895	ns	0.8654	ns	0.9994	ns	0.7472	*	0.0117	***	0.0002	****	<0.0001	****	<0.0001	ns	>0.9999
SAM2 vs. SAM5	ns	>0.9999	ns	>0.9999	ns	0.7853	ns	0.6878	****	<0.0001	ns	>0.9999	ns	0.9863	ns	>0.9999	ns	0.8978	ns	>0.9999
SAM2 vs. SAM6	ns	0.9797	ns	>0.9999	ns	>0.9999	****	<0.0001	****	<0.0001	****	<0.0001	****	<0.0001	****	<0.0001	****	<0.0001	****	<0.0001
SAM3 vs. SAM4	ns	0.0753	ns	0.5656	ns	0.9948	ns	0.0755	**	0.0044	***	0.0001	**	0.0054	ns	0.9912	****	<0.0001	ns	>0.9999
SAM3 vs. SAM5	ns	0.0863	ns	0.1438	ns	0.3826	ns	0.651	ns	0.8786	****	<0.0001	****	<0.0001	****	<0.0001	ns	0.8978	ns	>0.9999
SAM3 vs. SAM6	**	0.0013	ns	0.0747	ns	0.9976	ns	0.5898	ns	>0.9999	ns	0.8299	ns	0.9994	ns	0.7639	****	<0.0001	****	<0.0001
SAM4 vs. SAM5	ns	>0.9999	ns	0.9943	ns	0.0747	ns	0.9422	****	<0.0001	*	0.0178	**	0.0072	****	<0.0001	**	0.0075	ns	>0.9999
SAM4 vs. SAM6	ns	0.923	ns	0.968	ns	0.8411	***	0.0001	*	0.0123	*	0.0312	*	0.0323	ns	0.9954	ns	0.8791	****	<0.0001
SAM5 vs. SAM6	ns	0.9056	ns	>0.9999	ns	0.8139	**	0.0095	ns	0.7196	****	<0.0001	****	<0.0001	****	<0.0001	****	<0.0001	****	<0.0001
