# Supplementary figures and images for: It Takes Two to Make a Thing Go Right: Epistasis, Two-Component Response Systems, and Bacterial Adaptation
Source: Microorganisms. 2024 Sep 30;12(10):2000. doi: 10.3390/microorganisms12102000 (PMC11510482; doi:10.3390/microorganisms12102000)

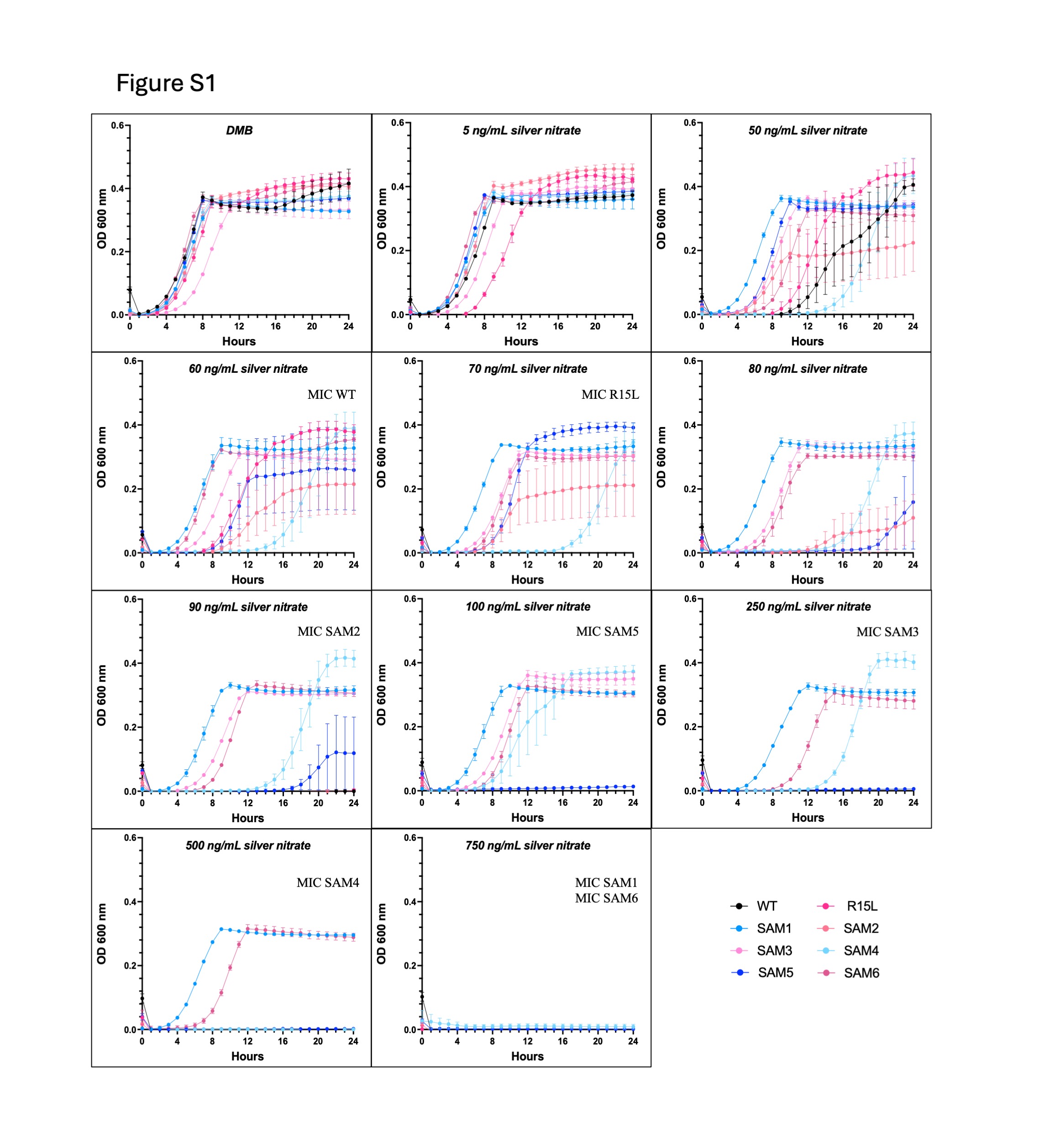

Supplement: Supplementary file 1 [file microorganisms-12-02000-s001.zip › FigureS1.jpeg]
